# Phenyl Bis-Sulfonamide Keap1-Nrf2 Protein–Protein Interaction Inhibitors with an Alternative Binding Mode

**DOI:** 10.1021/acs.jmedchem.2c00457

**Published:** 2022-05-12

**Authors:** Nikolaos Georgakopoulos, Sandeep Talapatra, Dina Dikovskaya, Sharadha Dayalan Naidu, Maureen Higgins, Jemma Gatliff, Aysel Ayhan, Roxani Nikoloudaki, Marjolein Schaap, Klara Valko, Farideh Javid, Albena T. Dinkova-Kostova, Frank Kozielski, Geoffrey Wells

**Affiliations:** † UCL School of Pharmacy, University College London, 29/39 Brunswick Square, London WC1N 1AX, U.K.; ‡ Stevenage Bioscience Catalyst, Keregen Therapeutics Ltd., Gunnels Wood Rd, Stevenage SG1 2FX, U.K.; § Jacqui Wood Cancer Centre, Division of Cellular Medicine, School of Medicine, University of Dundee, Dundee DD1 9SY, Scotland, U.K.; ∥ Bio-Mimetic Chromatography Consultancy, 17 Cabot Close, Stevenage SG2 0ES, U.K.; ⊥ Department of Pharmacy, University of Huddersfield, Queensgate, Huddersfield HD1 3DH, U.K.; # Department of Pharmacology and Molecular Sciences and Department of Medicine, School of Medicine, Johns Hopkins University, Baltimore, Maryland 21205, United States

## Abstract

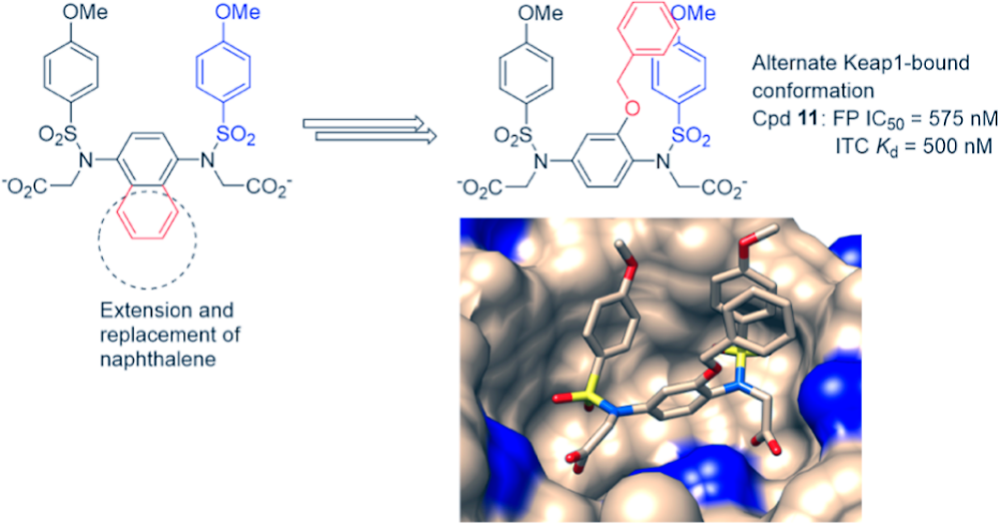

Inhibitors of Kelch-like ECH-associated protein 1 (Keap1) increase the activity of the transcription factor nuclear factor erythroid 2-related factor 2 (Nrf2) by stalling its ubiquitination and degradation. This enhances the expression of genes encoding proteins involved in drug detoxification, redox homeostasis, and mitochondrial function. Nrf2 activation offers a potential therapeutic approach for conditions including Alzheimer’s and Parkinson’s diseases, vascular inflammation, and chronic obstructive airway disease. Non-electrophilic Keap1-Nrf2 protein–protein interaction (PPI) inhibitors may have improved toxicity profiles and different pharmacological properties to cysteine-reactive electrophilic inhibitors. Here, we describe and characterize a series of phenyl bis-sulfonamide PPI inhibitors that bind to Keap1 at submicromolar concentrations. Structural studies reveal that the compounds bind to Keap1 in a distinct “peptidomimetic” conformation that resembles the Keap1-Nrf2 ETGE peptide complex. This is different to other small molecule Keap1-Nrf2 PPI inhibitors, including bicyclic aryl bis-sulfonamides, offering a starting point for new design approaches to Keap1 inhibitors.

## Introduction

The transcription factor Nrf2 (nuclear factor-erythroid 2-related factor 2) is a key regulator protein involved in adaptive responses to internal and external stress stimuli, particularly oxidative damage. Nrf2 augments the expression of a wide range of cyto-protective proteins by binding with high affinity to the antioxidant response elements that are located in their promoter gene regions. Gene products regulated by Nrf2 include proteins that maintain a reducing environment within cells, phase I and II metabolic enzymes, anti-inflammatory mediators, and proteins associated with general and selective types of autophagy.^1^

The transcriptional activity of Nrf2 is controlled by several regulatory mechanisms, among which the Keap1 (Kelch-like ECH associating protein 1)/Cullin3 system is the most extensively studied. Keap1 is a homodimeric protein that physically interacts through its Kelch domain with Nrf2 in a 2:1 stoichiometry.^2^ Mutagenesis and deletion analysis revealed that this interaction is mediated by two discrete regions within the Neh2 (Nrf2–ECH homology 2) domain of Nrf2 that consists of a low affinity ^29^DLG^31^ and a high affinity ^79^ETGE^82^ binding motif, respectively.^3,4^ This two-site recognition mechanism allows Keap1 to suppress the activity of Nrf2 by facilitating its ubiquitination and subsequent proteosomal degradation via a pendant Cul3-based Rbx1 (ring-box 1) E3 ubiquitin ligase complex. However, upon oxidative or electrophilic stress the repressor activity of Keap1 is impaired, allowing Nrf2 to evade degradation.^4^ The stabilization of Nrf2 under such conditions stems from the redox or covalent modification of Keap1 cysteine residues, which induces conformational changes in the structure of Keap1 that diminish its ubiquitin-facilitating activity.

Given its pivotal role in adjusting cellular defences under stress conditions and during hormesis, Nrf2 has been proposed to hold great promise as a drug target for the prevention or treatment of a wide range of pathological and chronic conditions, including but not limited to inflammatory conditions, neurodegenerative and autoimmune disorders and cardiovascular diseases.^1,5−7^ Compounds that inhibit Keap1 have been shown to enhance the transcriptional activity of Nrf2 and have therefore been sought extensively as potential therapeutic agents.

**Figure 1:**
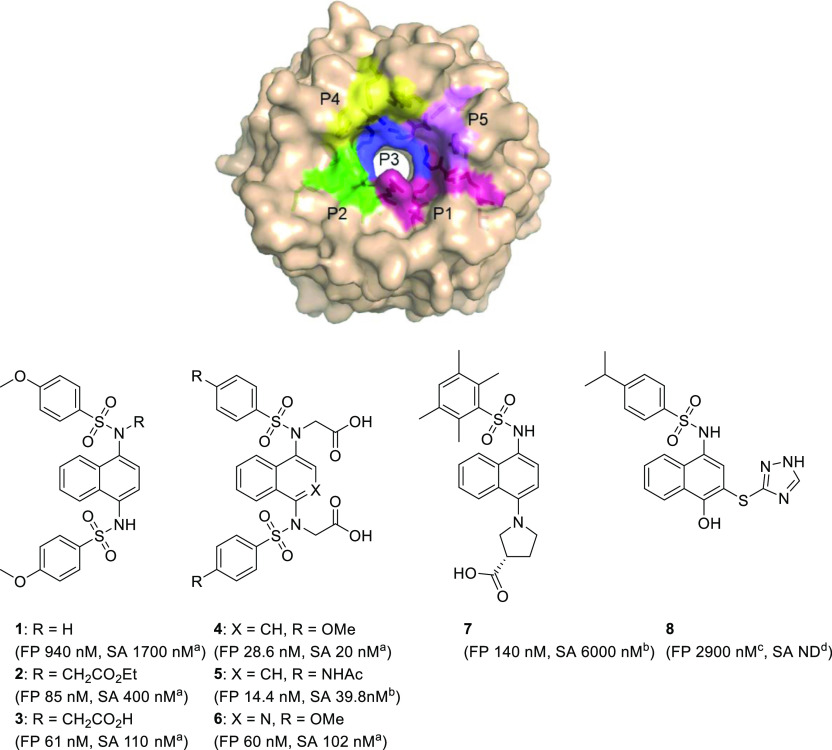
(A) Structure of the Keap1 Kelch domain (PDB entry 4IQK) shown as a surface representation, the sub-pockets of the Nrf2 binding site are shown: P1 (maroon residues 415, 461, 462, 478, 483, and 508), P2 (green residues, 363, 380, 381, and 414), P3 (blue residues, 364, 509, 556, 571, 602, and 603), P4 (yellow residues, 334, 572, and 577), and P5 (purple residues, 525, 530, and 555); (B) the structures of published Keap1-Nrf2 PPI inhibitors containing a naphthalene central ring system. FP values for compounds **1**–**8** are IC_50_s determined using FP; SA values are *K*_d_s from a secondary assay; (a) *K*_d_ determined using SPR assay; (b) *K*_d_ determined using ITC; (c) fluorescence anisotropy assay; and (d) not determined (DSF thermal shift = −5 °C).^13,14,17,18,24,27^

Most Nrf2 inducers are electrophiles that exert their effects by covalently modifying sensor Keap1 cysteines and subsequently, halting the ubiquitination and proteasomal targeting of the transcription factor. Given their highly reactive nature however, such agents generally demonstrate unspecific biological effects and interference with other redox sensitive signaling pathways. Direct inhibition of the Keap1-Nrf2 protein–protein interaction (PPI) is an alternative approach to inhibit the degradation of Nrf2 in a reversible and non-covalent manner. We and others have described the development of peptides based on the ETGE and DLG binding motifs of Nrf2 that compete with the transcription factor for binding to the Kelch domain of Keap1.^8−11^ More recently, a series of small molecule modulators of the Keap1-Nrf2 PPI have been described,^12−22^ and Keap1 was the focus of an ultralarge virtual screen of more than 1 billion compounds.^23^ Although in the latter case promising levels of both in vitro and cellular potency have been achieved, there is a relatively limited degree of structural diversity among the different classes of PPI inhibitors reported. A number of PPI inhibitors described to date share a common privileged scaffold comprising a bicyclic ring system, usually a naphthalene ring flanked by one or in most cases two benzenesulfonamide moieties (Figure 1). According to the available crystallographic and molecular modeling data, the bicyclic ring occupies the P3 sub-pocket of the Keap1 Kelch binding site and enables the projection of the benzenesulfonamide group(s) into the P4 and/or P5 sub-pockets. Previous attempts to replace the naphthalene group with a single ring system have resulted in a reduced Keap1 binding affinity, highlighting the favorable Keap1 binding properties of this scaffold.^14,24^ These can be attributed to the formation of a network of π–cation and π–π stacking interactions of the naphthalene ring with the guanidino group of Arg415 and the benzenesulfonamide moieties with the aromatic side chains of Tyr334, Tyr572 and Tyr525. Recent studies have revealed that, in contrast to electrophiles, which impair the substrate adaptor activity of Keap1, leading to the accumulation of newly synthesized Nrf2, such non-electrophilic PPI inhibitors disrupt the binding of Keap1 to the DLG motif of Nrf2 in preference to Keap1-ETGE binding.^25,26^

Here, we report the development and biological characterization of a novel Keap1-interactive scaffold identified through a structure-based ligand design approach. Although structurally similar to previously described phenyl bis-sulfonamides such as **9**, several of the new analogues exhibit sub-micromolar affinity for the Kelch domain of Keap1 as determined by fluorescence polarization (FP) and isothermal titration calorimetry (ITC) analyses.^14^ Despite the presence of two carboxylate groups, selected compounds interact with Keap1 and induce the transcriptional activity of Nrf2 in cells at micromolar concentrations. The improved binding affinity and inducer activity may be explained by a high-resolution co-crystal structure of analogue **11** bound to the Keap1 Kelch domain that reveals a new and unexpected binding mode that closely resembles the Keap1-Nrf2 ETGE peptide complex and is distinct from the binding mode of any other known aryl bis-sulfonamides, which preferentially disrupts the binding of Keap1 to Nrf2-DLG.^25^ Thus, the new series of Keap1-Nrf2 PPI provide an alternative starting point for future optimization that exploits this new binding mode.

## Results and Discussion

### Molecular Modeling and Ligand Design

**Figure 2:**
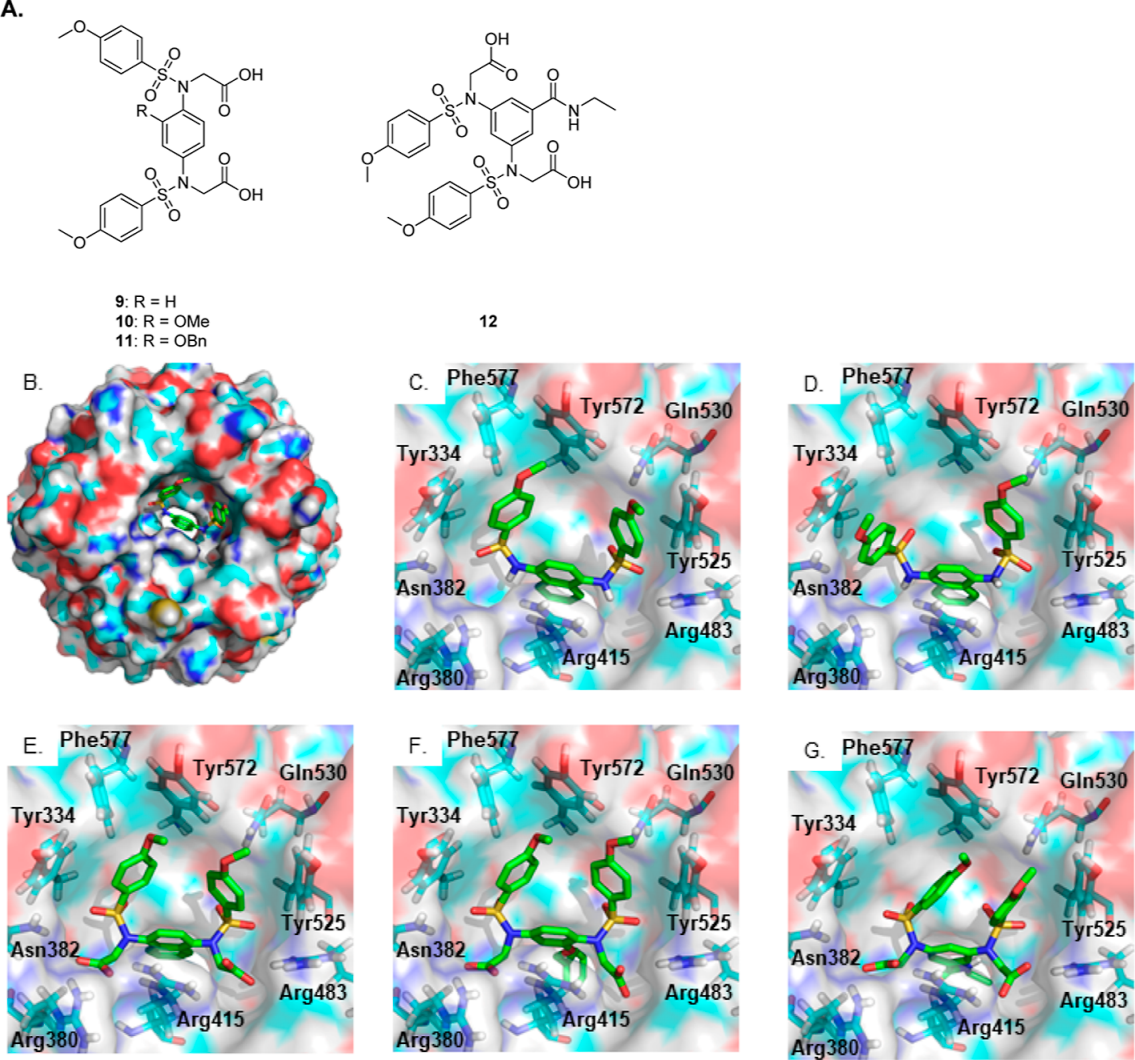
(A) Structures of compounds **9**–**12**; (B,C) co-crystallized structure of **1** bound to the Keap1 Kelch domain (PDB entry 4IQK); (D–G) docked conformations of compounds **1**, **4**, **11**, and **12**, respectively, bound to the corresponding 4IQK Keap1 Kelch domain.

Although several studies describing the structure–activity relationships (SARs) around the phenylsulfonamide^14,17^ and acetate^28^ portions of **4** have been published, there is less information about the structural requirements for the central ring system of this scaffold. Replacement of the naphthalene with related bicyclic heterocycles such as isoquinolines, for example, **6** gives ligands of similar binding affinity and improved properties, but other heterocycles had reduced binding affinity.^24^ Recent reports showed that replacement of the naphthalene core of **4** with a phenyl group reduces binding affinity; however, **9** retains only low micromolar affinity for Keap1 that can be moderately improved by introducing a methoxy substitution at the 2-position of the phenyl ring (Figure 2A, compound **10**).^14,24^ We carried out a molecular docking study using the Keap1 Kelch domain structure derived from the co-crystal structure with **1** (PDB entry 4IQK, Figure 2B,C). We confirmed that redocking **1** into the vacant Kelch binding pocket gave a comparable bound conformation and that **4** docked in the expected manner (Figure 2D,E, respectively) to make additional polar contacts with the arginine triad, residues 380, 415, and 483. Next, we docked the ligands **11** and **12** in an analogous manner (Figure 2F,G). These new ligands have a central phenyl ring and either a 1,4- or 1,3-substitution pattern for the sulfonamide moieties. The docked conformations suggested that the bis-sulfonamide was positioned in a similar manner to those of **1** and **4** and that the respective benzyloxy and carboxamide substituents of **11** and **12** project into the central channel through the β-propeller structure, indicating that they may occupy the P3 sub-pocket of Keap1 more effectively. These encouraging preliminary data prompted the synthesis of a series of analogues based on the selected scaffolds with the aim of determining the structural requirements for interaction with the Keap1 Kelch domain central pore.

### Chemical Synthesis

**Scheme 1:**
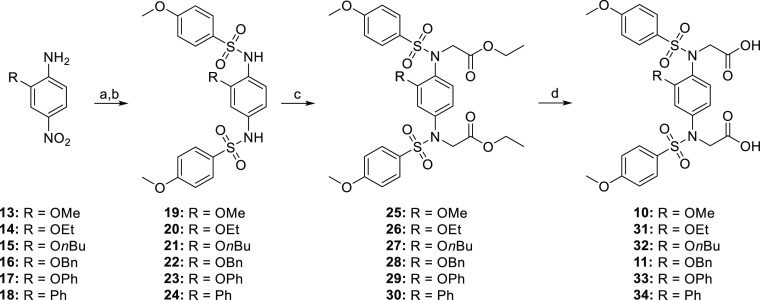
Synthetic Route to Compounds **10**–**11** and **31**–**34** Reagents and conditions: (a) Fe, AcOH, EtOH/H_2_O, 65 °C, 2 h or SnCl_2_·2H_2_O, EtOH, 60 °C, 2 h; (b) 4-methoxybenzenesulfonyl chloride, pyridine, DMAP, DCM, rt, o/n; (c) ethyl bromoacetate, K_2_CO_3_, DMF, rt, 6 h; and (d) 1 N NaOH, THF//MeOH (1:1), rt, 2 h.

The synthesis of compounds **31**–**36** was performed following the general procedure described in Scheme 1. The nitro groups of **13**–**18** were reduced with either Fe/AcOH or SnCl_2_ and the resulting intermediates were reacted with an excess of 4-methoxybenzenesulfonyl chloride and pyridine to afford the bis-sulfonamides **19**–**24**. N-alkylation with ethyl bromoacetate and K_2_CO_3_ followed by saponification of the resulting ethyl esters **25**–**30** gave the bis-acids **10**–**11** and **31**–**34** in reasonable overall yields.

**Scheme 2:**
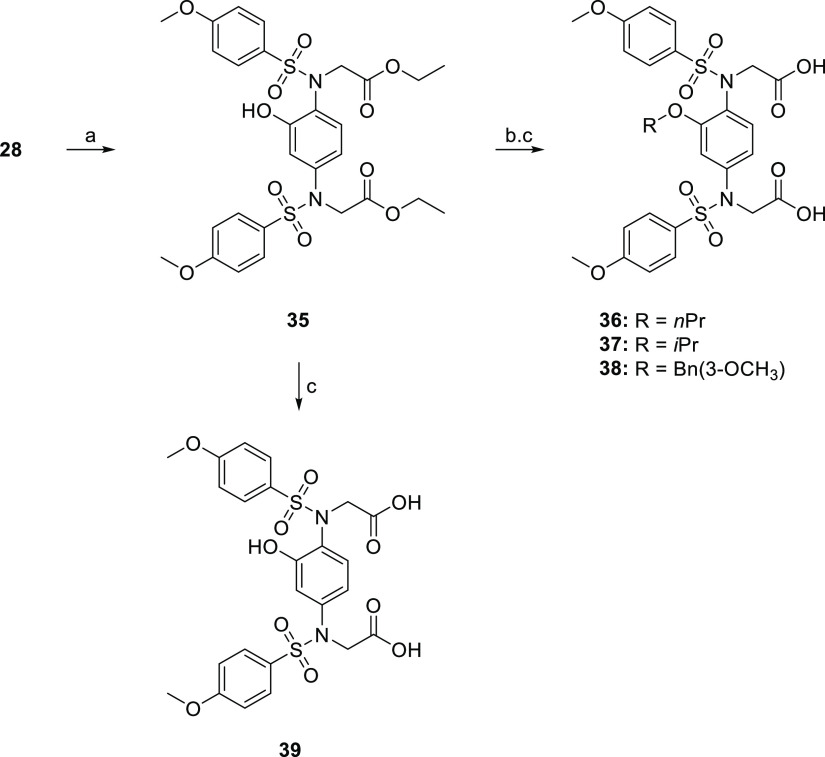
Synthetic Route to Compounds **35**–**39** Reagents and conditions: (a) triethylsilane, 5% Pd/C, EtOAc/EtOH (9:1 v/v), rt, 2 h, 61%; (b) R–X, K_2_CO_3_, acetone, rt, o/n; and (c) 1 N NaOH, THF//MeOH (1:1), rt, 2 h.

To further supplement our compound library, we prepared compounds **36**–**39** from a common precursor (compound **28**) following the alternative and shorter synthetic sequence outlined in Scheme 2. The benzyl ether of **28** was removed by catalytic transfer hydrogenation, affording the phenol **35** in 61% yield. **35** was subsequently subjected to saponification to furnish **39**, while the remaining analogues **36**–**39** were prepared by the alkylation of the phenol **35** with appropriate halides, followed by hydrolysis of the ethyl ester protecting groups.

**Scheme 3:**
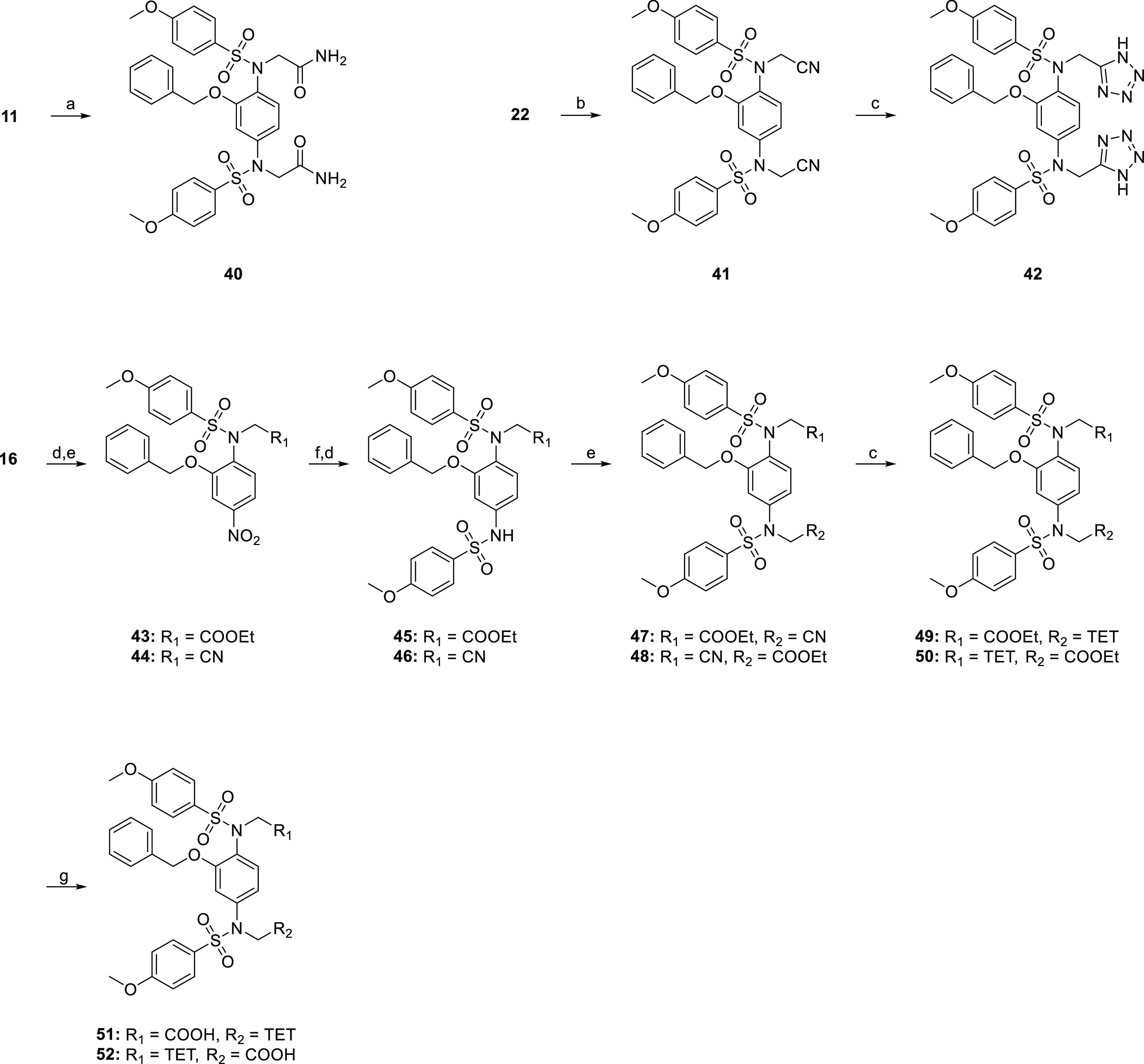
Synthetic Route to Compounds **40**–**42**, **51**, and **52** Reagents and conditions: (a) Boc_2_O, (NH_4_)_2_CO_3_, pyridine, DMF, Ar, 0 °C to rt, 18 h, 74%; (b) bromoacetonitrile, K_2_CO_3_, DMF, rt, 8 h, 59%; (c) NaN_3_, NH_4_Cl, DMF, 100 °C, o/n; (d) 4-methoxybenzenesulfonyl chloride, DCM, pyridine, rt, o/n; (e) ethyl bromoacetate, K_2_CO_3_, DMF, rt, 8 h; (f) SnCl_2_·2H_2_O, EtOH, 65 °C, 1 h; and (g) 1 N NaOH, THF/MeOH (1:1 v/v), 2 h.

Compounds bearing carboxylate bioisosteric replacements were prepared according to the synthetic procedures shown in Scheme 3. The bis-amide **40** was synthesized from **11** by treatment with Boc_2_O and ammonium carbonate in DMF/pyridine. N-alkylation of **22** with bromoacetonitrile gave **41** and then cycloaddition with NaN_3_ afforded the bis-1*H*-tetrazole **42**. The remaining acid/1*H*-tetrazole analogues **51** and **52** were prepared from **16** via sequential assembly of the tertiary sulfonamide moiety on each side of the central phenyl ring, followed by [2 + 3] cycloaddition with sodium azide and subsequent ethyl ester hydrolysis.

**Scheme 4:**
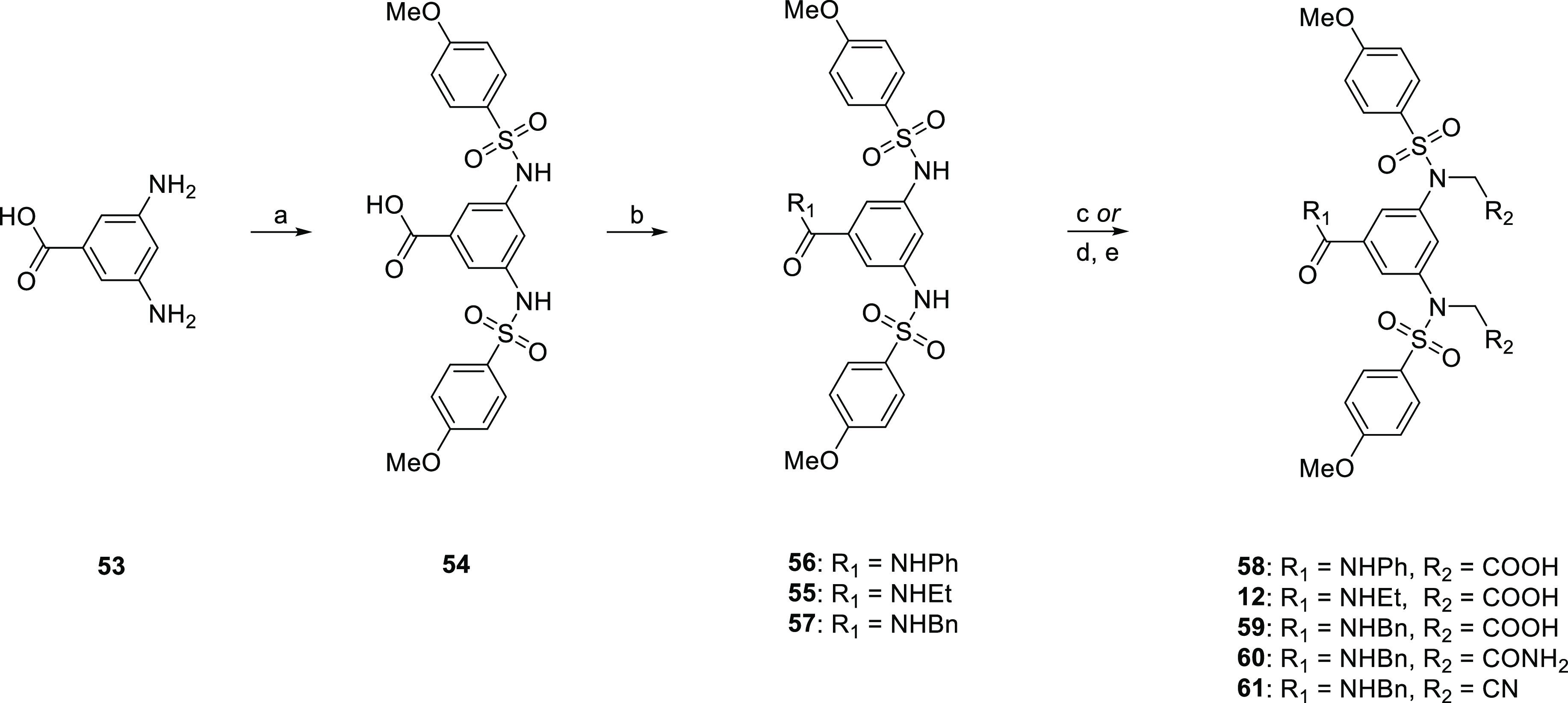
Synthetic Route to Compounds **12** and **58**–**61** Reagents and conditions: (a) 4-R_1_-PhSO_2_Cl, pyridine, DMAP, DMF, 100 °C, o/n; (b) amine, EDCI·HCl, DMAP, DMF, o/n; (c) bromoacetonitrile or bromoacetamide, K_2_CO_3_, DMF, rt, o/n; (d) ethyl bromoacetate, K_2_CO_3_, DMF, rt, o/n; and (e) 1 N NaOH, THF/MeOH (1:1 v/v), o/n.

The related 3,5-bis-sulfonamide-*N*-substituted-benzamides **12** and **58**–**61** were prepared from the readily available 3,5-diaminobenzoic acid following a similar synthetic procedure (Scheme 4). Sulfonylation of **53** using our standard conditions, followed by amidation with EDCI·HCl and DMAP afforded intermediates **54** and **55**–**57**, respectively. Compounds **12** and **58**–**61** were synthesized by nucleophilic substitutions using the appropriate alkyl halides, followed by the removal of the protecting ethyl ester groups for compounds **12** and **58**–**59**.

### Structure–Activity Relationships

The compounds were screened for their ability to disrupt the interaction of the Keap1 Kelch domain with a fluorescent peptide derived from the high affinity ETGE binding motif of Nrf2 (FITC-β-DEETGEF-OH) in an FP competition assay.^8^ Generally, a variety of substitutions were tolerated at the 2-position of the central phenyl ring (Table 1). The phenol **39** demonstrated reasonable inhibition of the Keap1-Nrf2 PPI, while capping the phenolic OH with a methyl group (compound **10**) did not affect the binding affinity. Replacing the 2-methoxy substitution with an ethoxy (compound **31**) led to improved activity; however, increasing the size of the 2-alkoxy substituent from ethoxy to *n*-propyloxy (compound **36**) was not beneficial for activity. A complete loss of binding affinity was recorded for branched isopropyloxy **37**, presumably due to the introduction of an unfavorable steric clash with the Keap1 binding pocket. Interestingly, a compromise in activity was achieved by further extending the length of the 2-alkoxy moiety (*n*-butyl, compound **32**), suggesting that introduction of bulkier substituents at this position could lead to an improved binding profile. In support of this notion, the benzyloxy analogue **11** demonstrated submicromolar activity in the FP assay with an IC_50_ value of 0.575 μM. Removing the benzylic methylene bridge of **11** (compound **33**) decreased the activity by threefold, while further simplification of the structure by replacing the ether substituent with a phenyl ring (compound **34**) further diminished binding affinity. On the other hand, functionalization of the benzyloxy group by introducing a *meta*-methoxy substitution was tolerated (compound **38**) and resulted in a modest loss of binding activity.

**Table 1:**
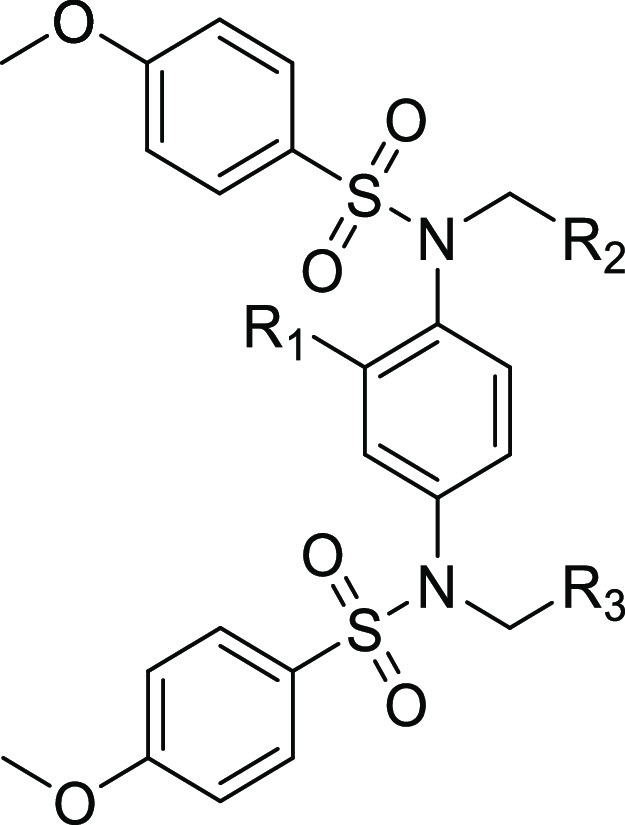
SARs and Physicochemical Properties for Compounds **10**–**11**, **31**–**34**, **36**–**42**, **51**, and **52**

compd	R_1_	R_2_	R_3_	FP IC50 (μM)	DSF (Δ*T*_i_ °C)^a^	NQO1-fold induction at 10 μM^b^
**10**	OMe	CO_2_H	CO_2_H	1.84 ± 0.22	2.4 ± 0.3	0.94 ± 0.13
**31**	OEt	CO_2_H	CO_2_H	0.815 ± 0.060	2.4 ± 0.3	1.65 ± 0.22
**32**	O*n*Bu	CO_2_H	CO_2_H	0.677 ± 0.059	3.1 ± 0.2	1.66 ± 0.08
**11**	OBn	CO_2_H	CO_2_H	0.575 ± 0.072	3.3 ± 0.4	1.71 ± 0.09
**33**	OPh	CO_2_H	CO_2_H	1.50 ± 0.22	3.1 ± 0.4	0.92 ± 0.11
**34**	Ph	CO_2_H	CO_2_H	51%^c^	0.2 ± 0.3	1.08 ± 0.04
**36**	O*n*Pr	CO_2_H	CO_2_H	1.19 ± 0.23	2.9 ± 0.2	1.29 ± 0.22
**37**	O*i*Pr	CO_2_H	CO_2_H	<25%^c^	0.1 ± 0.3	1.09 ± 0.07
**38**	OBn(3-OMe)	CO_2_H	CO_2_H	0.805 ± 0.021	3.4 ± 0.3	1.84 ± 0.05
**39**	OH	CO_2_H	CO_2_H	1.11 ± 0.32	1.2 ± 0.3	1.00 ± 0.06
**40**	OBn	CONH_2_	CONH_2_	<25%^c^	ND^d^	1.19 ± 0.07
**41**	OBn	CN	CN	<25%^c^	0.1 ± 0.2	1.02 ± 0.05
**42**	OBn	TET	TET	37%^c^	ND^d^	1.06 ± 0.10
**51**	OBn	CO_2_H	TET	2.93 ± 0.33	ND^d^	1.06 ± 0.04
**52**	OBn	TET	CO_2_H	56%^c^	ND^d^	1.10 ± 0.04

a Inflection temperature (*T*_i_) for Keap1 protein + vehicle = 61.3 ± 0.1 °C, Δ*T*_i_ for cpd **4** = 20.0 ± 0.3 °C.

b Fold-induction of NQO1 enzymatic activity relative to the DMSO control after 24 h inhibitor treatment, cpd **4** = 2.85 ± 0.14 fold.

c Percentage inhibition at 10 μM concentration of inhibitor.

d Not determined.

To complement our SAR model, we then explored the effect of carboxylate isosteric transformations on binding activity. For this purpose, we selected **11** as a model scaffold due to its superior potency compared to other compounds of this series. Replacing both carboxylate groups of **11** with carboxamides (compound **40**) or nitriles (compound **41**) abolished binding affinity, possibly due to the inability of these groups to form the necessary salt bridging interactions with the triad of positively charged arginine residues that are located near to the P1 and P2 sub-pockets of the Keap1 Kelch binding site. Similar results were obtained for the bis-1*H*-tetrazole **42**, although some marginal inhibitory activity was detected at the highest concentration screened (10 μM). As expected, substituting only one carboxylic acid of **11** for a 1*H*-tetrazole (compounds **51** and **52**) was better tolerated; however, activity was reduced by at least sixfold in both cases.

The 1,3-bis-sulfonamide benzamides **12** and **58**–**61** showed a consistently weaker binding profile in the FP assay compared to the previous examples (Table S1). The *N*-phenyl **58** and *N*-benzyl **59** analogues demonstrated modest activity at 10 μM, causing approximately 50% inhibition of the interaction between the Keap1 Kelch protein and the fluorescent peptide. On the other hand, the *N*-ethyl **12** was unable to markedly inhibit the FP signal at the same concentration. Analogues having both carboxylates substituted for carboxamides (compound **60**) or nitriles (compound **61**) did not show activity at 10 μM, results that are consistent with the SAR obtained from the 2-benzyloxy derivatives **40** and **41**.

The binding affinity of compounds was confirmed in a secondary orthogonal differential scanning fluorimetry (DSF) assay at a fixed concentration of 10 μM using a label-less adaptation of a published procedure in a Nanotemper Tycho instrument that measures changes in the ratio of protein tyrosine and tryptophan intrinsic fluorescence with temperature.^20^ The fluorescence ratio inflection temperature (*T*_i_) for the Keap1 Kelch protein was estimated to be 61.3 ± 0.1 °C using this method. Compound **4** was used as the positive control and gave a Ti of 81.3 ± 0.3 °C, corresponding to an induced shift in the *T*_i_ (Δ*T*_i_) of 20 °C. The Keap1 Kelch melting curves were shifted to a smaller extent (Δ*T*_i_ of ∼2.4–3.4 °C) relative to the positive control in the presence of compounds **10**, **11**, **31**–**33**, **36**, and **38**, confirming their direct interaction with the protein, while **34**, **37**, **39**, and **41** gave smaller Δ*T*_i_ values (Δ*T*_i_ ≈ 0.1–1.2 °C) consistent with their lower activity in the FP assays.

To further characterize the binding profile of the series, we carried out ITC experiments. Compound **11** had an estimated *K*_d_ of 0.500 ± 0.057 μM and compounds **31**, **32**, **36**, and **38** had *K*_d_ values in the range 0.429–0.638 μM (Figure S1), which were comparable with the IC_50_ values recorded in the FP assay (Table 1). The reference compound **4** had a *K*_d_ of 0.039 ± 0.010 μM, consistent with previous observations (Figure 1).

### Crystal Structure of the Keap1–Compound **11** Complex

**Figure 3:**
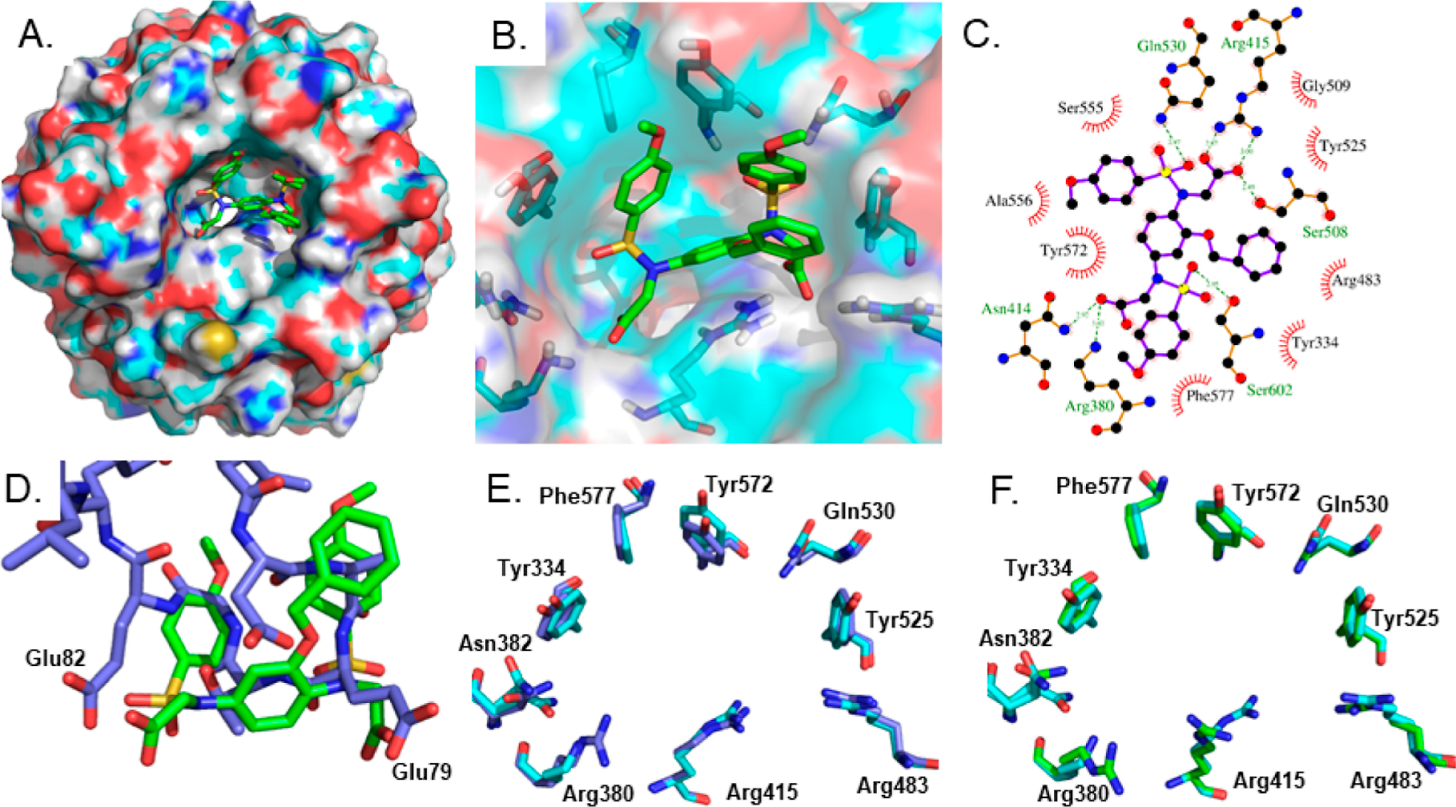
(A–C) Bound conformation of compound **11** (green in A and B, purple in C) co-crystallized with the Keap1 Kelch domain; (D) overlay of the bound conformation of **11** and the ETGE peptide bound to Keap1 (PDB entry 2FLU);^29^ (E) comparison of the Keap1 Kelch domain binding pocket in the compound **11** co-crystal structure (cyan) and the 2FLU ETGE peptide co-crystal (blue); (F) comparison of the Keap1 Kelch domain binding pocket in the compound **11** co-crystal structure (cyan) and the compound **1** co-crystal structure (green).^19^

The overall structure of the Keap1 Kelch domain in complex with compound **11** was solved at a resolution of 1.75 Å (PDB entry 6HWS). The Keap1 Kelch domain adopts a six-bladed β-propeller structure with a central hollow channel.^30^ Compound **11** binds to the Nrf2 binding region which is located over the central pore through the protein domain. The central phenyl ring of compound **11** occupies the P3 sub-pocket of the Keap1 Kelch binding site, forming a π–cation interaction with the positively charged guanidino group of Arg415 (Figure 3A,B). Similarly to the naphthalene-based inhibitors reported previously,^17,19^ the flanking 4-methoxybenzenesulfonamide moieties form π–π stacking interactions with the aromatic side chains of Tyr334, Tyr572, Phe577, and Tyr525 of the P4 and P5 sub-pockets, whereas one of the sulfonamide oxygen atoms forms a hydrogen bond with the carboxamide side chain group of Gln530 (Figure 3C). The two acetate side chains of **11** are projected toward the P1 and P2 sub-pockets with the respective carboxylate groups participating in a network of hydrogen bonding and charge–charge interactions with Arg415, Ser508, Asn414, and Arg380. Interestingly, the benzyloxy phenyl group stabilizes the bound conformation of **11** by forming a face-to-face π–π stacking interaction with the 4-methoxybenzene ring of the adjacent sulfonamide side chain, while it also participates in an edge-to-face cation−π stacking with Arg483 (Figure 3B,C).

The bound conformation of **11** contrasts with the predicted docked conformation (Figure 2F) in which the benzyloxy substituent occupies the central channel through the protein and instead has a greater similarity to that of ETGE peptide-bound forms of the Keap1 Kelch domain (e.g., PBD entry 2FLU)^29^ in which the binding pocket is adapted to fit the peptide. With respect to the ligand, one of the carboxylic acids of **11** and the ETGE peptide glutamate residue equivalent to Glu79 overlay (Figure 3D, lower right) and the peptide backbone overlays with the adjacent sulfonamide motif. The Keap1 binding pocket residues are also in a very similar orientation in the **11**-bound and peptide-bound structure (Figure 3E), particularly with regard to the Arg415 residue, which is usually rotated in small molecule-bound structures to expose the central channel through the β-propeller structure (e.g., the complex with **1**, PDB entry 4IQK, Figure 3F).

### Cell-Based Experiments

**Figure 4:**
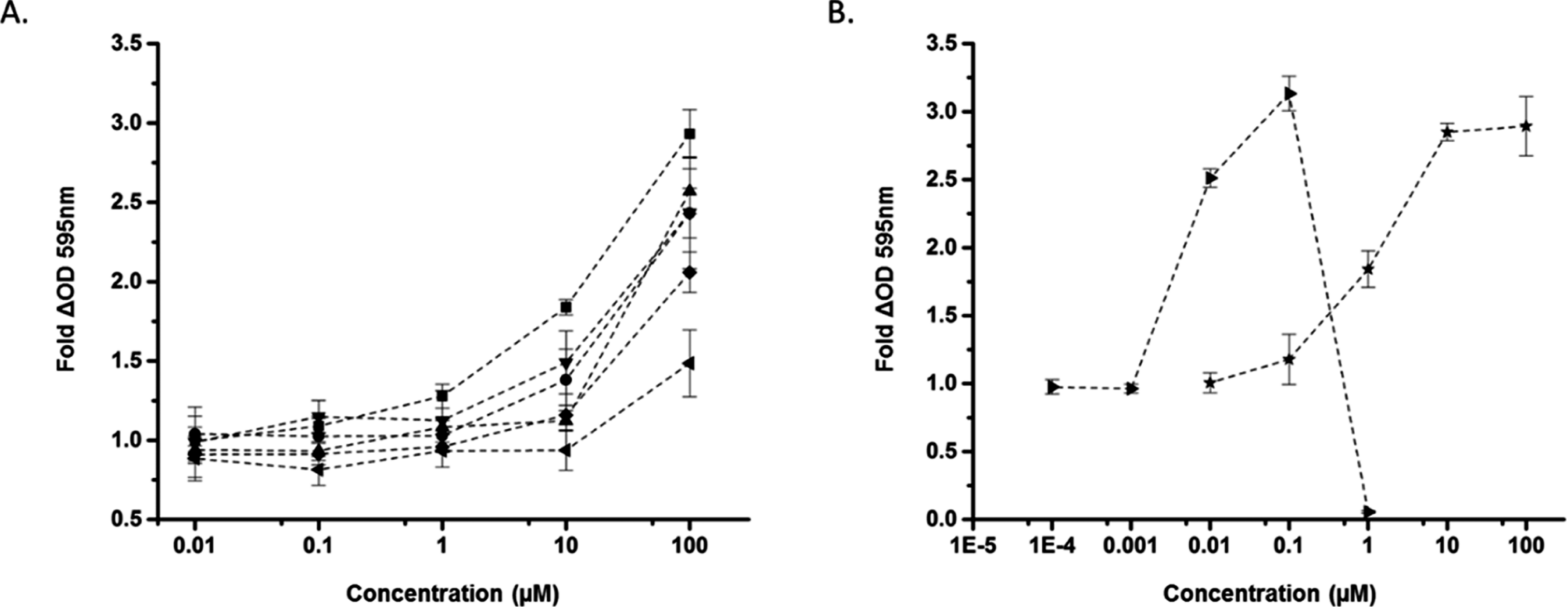
Dose-dependent induction of NQO1 [NAD(P)H-dependent quinone oxidoreductase-1] by selected (A) Keap1-Nrf2 PPI inhibitors: **10** (◀), **11** (▼), **31** (▲), **32** (●), **36** (⧫), and **38** (■) and (B) electrophilic Nrf2 activators: DMF (★) and CDDO-Me (▶).

**Figure 5:**
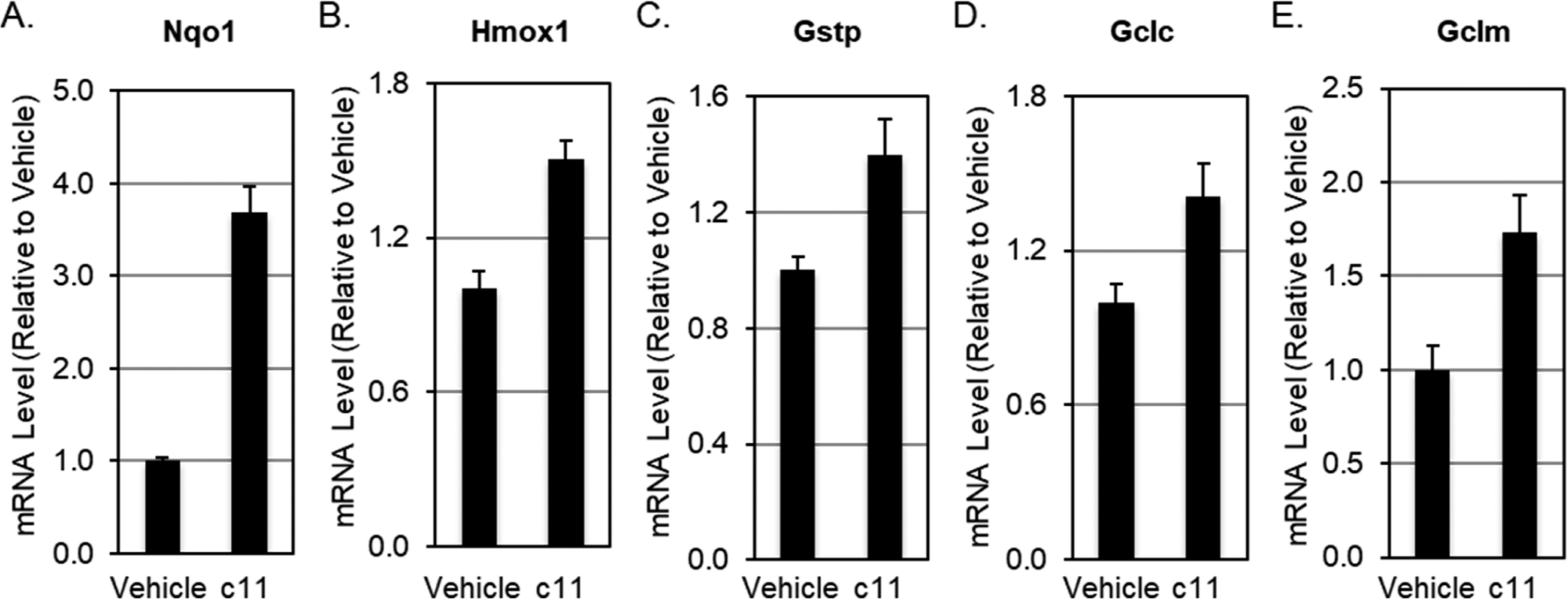
Induction of the Nrf2-transcriptional targets Nqo1 (A), Hmox1 (B), Gstp (C), Gclc (D), and Gclc (E) in Hepa1c1c7 cells (*n* = 3) by compound **11** (50 μM). The data are represented graphically as mean values ± 1 standard deviation (SD). Student’s *t*-test was used to test for statistical significance, and in all cases, the difference in gene expression between vehicle- and compound **11**-treated cells was statistically significant (*p* < 0.05).

The ability of compounds of this series to induce the transcriptional activity of Nrf2 was evaluated in Hepa1c1c7 mouse hepatoma cells using the colorimetric NAD(P)H quinone oxidoreductase 1 (NQO1) enzyme activity assay, an assay that is widely used to characterize the biological activity of Nrf2 inducers.^12,31,32^ We initially screened the compounds at a fixed dose of 10 μM (Table 1). This preliminary screening gave encouraging results as it revealed a good correlation between the in vitro and cell-based activities of the series. Compounds **31**, **32**, **11**, and **38** retained a good level of potency in cells, inducing the enzymatic activity of NQO1 by more than 1.5-fold relative to the DMSO control at 10 μM. On the other hand, compounds with a less tight binding to Keap1 failed to cause a marked induction of NQO1 at the same concentration. Motivated by these results, we carried out concentration–response studies with selected candidates in order to gain a more detailed understanding of the cellular activity profile of the series. As shown in Figure 4, the NQO1 activity was generally induced in a dose-dependent manner after a 24 h treatment by all compounds screened. The methoxy-containing compound **10** was the least potent among the analogues tested, displaying only marginal cellular activity (1.4-fold induction) at 100 μM, results that are consistent with its weak binding affinity for Keap1. On the other hand, **38** showed the most promising cellular activity profile, causing a reasonable increase in NQO1 (1.5-fold to 2-fold) at low micromolar concentrations that reaches a threefold induction at the highest concentration tested (100 μM). The maximal NQO1 activation induced by **38** is similar to that observed for the positive controls bardoxolone methyl (CDDO-Me) and dimethyl fumarate (DMF); however, the latter compounds are more active at lower concentrations. In contrast to CDDO-Me that was cytotoxic at concentrations higher than 1 μM, none of the phenyl ethers showed signs of cytotoxicity in this assay up to a final concentration of 100 μM. This observation was supported by cytotoxicity assays carried out in ARPE19 retinal pigment epithelial cells.^33^ We demonstrated that **11** was not cytotoxic at concentrations up to 100 μM. Under the same conditions, the corresponding naphthalene bis-sulfonamide **4** showed a small reduction in cell viability (70% cell viability @ 100 μM) and sulforaphane had an IC_50_ of ∼10 μM, consistent with its behavior in other cell lines (Table S2).^12^ Based on these data, compound **11** was evaluated using real-time quantitative PCR to confirm the induction of NQO1 that was observed in the enzymatic assays in mouse Hepa1c1c7 cells. After 16 h of exposure, an increase in the expression of NQO1 was observed (Figure 5A; consistent with the NQO1 enzymatic assay results). The increase in mRNA levels of other Nrf2 target genes including heme oxygenase 1 (Hmox1), glutathione *S*-transferase P (GstP), and glutamate-cysteine ligase catalytic (Gclc) and modulatory (Gclm) subunits (Figure 5B–E) indicated that the effect was not limited to NQO1.

In a proof of principle study, target engagement by compound **11** was further supported by use of the cellular thermal shift assay (CETSA).^34^ Lysates of human promyelocytic leukemia HL-60 cells were incubated with compound **11** for 1 h at 37 °C and then subjected to a temperature gradient. Immunoblotting analysis of the protein levels of Keap1 in the soluble fractions after the removal of heat-induced aggregates showed that the thermal stability of Keap1 was increased by the treatment with compound **11** (Figure S2), indicating that the compound binds to endogenous human Keap1 in the cellular environment.

The activities that we describe are consistent with inhibition of the PPI between Keap1 and Nrf2 and the subsequent upregulation of Nrf2 target genes. However, as with other studies of Keap1 inhibitors, we cannot rule out the possibility that the compounds have other biological activities that we have not determined in our assays.

### Physicochemical Properties

Given the promising results obtained from our SAR studies, we sought to further characterize this novel series of Keap1-Nrf2 PPI inhibitors by determining their aqueous solubility and transmembrane permeability. Solubility measurements were carried out at pH 7.4 using the Multiscreen HTS-PCF filter plates (Merck Millipore, MSSLBPC10), while transmembrane permeability was evaluated using the parallel artificial membrane permeation assay (PAMPA).^35^ Generally, the solubility of the series was good and within the range of 250–350 μg/mL (Table S3), which is comparable to the reported solubility values for the naphthalene-containing compound **4**. The highest-affinity analogue **11** had an aqueous solubility of 348 μg/mL, while modifications on the ether portion of the scaffold did not cause significant changes. On the other hand, bioisosteric replacements of both carboxylate groups of **11** with nitriles, amides, or 1*H*-tetrazoles reduced solubility by more than twofold. As expected, compounds of this chemical series were found to have a relatively low permeability at pH 7.4 due to the presence of the two ionizable carboxylate groups (Table S4). Modifications to the ether moiety of the scaffold had minimal effects on the permeability of the series. Similarly, replacing either carboxylic acid group with a 1*H*-tetrazole did not cause major changes in the log *Pe* and led to a similar degree of passive diffusion. Overall, compound **38** demonstrated the most promising combination of physicochemical properties among the compounds screened, which could explain its improved cellular activity profile.

These data prompted us to further investigate the potential of **38** as a lead molecule for further optimization by evaluating a series of its physicochemical and pharmaceutical properties using high-performance liquid chromatography (HPLC) methods.^36^ Chromatographic approaches have been widely applied to drug discovery for the determination of lipophilicity and biomimetic properties of preclinical drug candidates. For example, the chromatographic hydrophobicity index (CHI) provides an alternative approach to determine the lipophilicity of compounds that is based on their retention time in reversed-phase HPLC columns. CHI values can be converted into the log *D* scale (CHI log *D*) to enable comparisons with data obtained using the octanol–water partition coefficient method. Compound **38** demonstrated good lipophilicity in our HPLC measurements that was consistently higher compared to the naphthalene **4** in different pH environments (Table 2).

**Table 2:** Comparison of Physicochemical and Pharmaceutical Properties of Compounds **38** and **4**

compd	CHI log *D* 2.0	CHI log *D* 7.4	CHI log *D* 10.5	IAM binding log *K*(IAM)	*V*_d_ (L/kg)	DE_max_ (%)	HSA binding (%)	AGP binding (%)
**4**	2.75	0.57	0.61	1.64	0.01	0.94	>99.9	20.3
**38**	3.15	0.85	0.88	1.83	0.09	4.55	99.1	47.7

The improved hydrophobic character of **38** translated into an increased immobilized artificial membrane (IAM) binding, which represents the affinity of ligands for phospholipids and thus relates to permeability through the main physiological barriers. These results are consistent with the improved in vitro binding affinity to cell-based activity ratio of compound **38** relative to **4** (Table 1, Figure 1. We also carried out HPLC biomimetic experiments using stationary phases containing immobilized human serum albumin (HSA) and α-glycoprotein (AGP) to predict the binding of the compounds to the respective plasma proteins. Our results suggest that the two molecules have a relatively different plasma protein binding profile. In particular, naphthalene **4** demonstrated a very tight binding to HSA, as indicated by its apparent infinite retention inside the HSA-coated HPLC column, while **38** bound to HSA more weakly. The binding profiles of **38** and **4** to the AGP were also different, with the two compounds showing 47.7 and 20.3% binding, respectively. Compound **38** demonstrated a more than fourfold improvement in maximum drug efficiency percentage (DE_max_)^37^ relative to the naphthalene **4**, which predicts an improvement in free plasma concentration. Similarly, the volume of distribution (*V*_d_)^38^ of **38** is predicted to be larger than that of **4**. Optimizing the physicochemical properties of the compounds is relevant to their cellular permeability, which could be improved and also to the potential utility of the compounds in interventions in neurological conditions where the role of Nrf2 induction has a number of promising applications.^1,7,39,40^

## Conclusions

**Figure 6:**
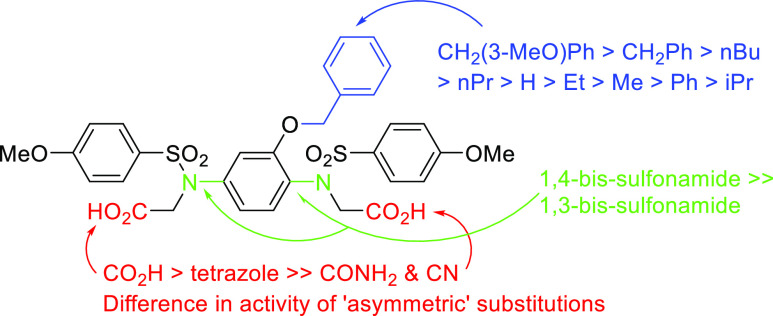
SARs for compounds **10**–**12**, **31**–**42**, **51**–**52**, and **58**–**61**.

In this article we present the design and synthesis of a series of phenyl bis-sulfonamide inhibitors of Keap1. Compound **11** binds to a Keap1 conformation that is comparable to its peptide bound form which distinguishes it from all other Keap1 PPI-inhibitory small molecules to date. The SARs for the compound series (Figure 6) demonstrate the effects of substitution at the newly introduced ether substituent, the carboxylate moieties and the positioning of the sulfonamide moieties. Together with the structural data from compound **11**, this suggests that the core structure could be further modified, for example, by changes to the linker length for the acid motifs, exploration of additional carboxylate isosteres, replacement of the benzyloxy substituent with substituted analogues or heterocycles and modifications to the sulfonamide substituents. The current compounds bind to Keap1 with mid-nanomolar binding affinity, interact with Keap1 in cells, and increase the expression of Nrf2 target genes. Compound **11** lacked cytotoxic activity in ARPE19 cells. The structural characterization of the unique bound conformation of the ligands and the assessment of their physicochemical properties and liabilities provide a basis for further optimization of this new subtype of the Keap1 inhibitor.

## Experimental Section

### Chemistry Methods

#### General

All anhydrous solvents and reagents were purchased from commercial suppliers and used without further purification.

^1^H NMR spectra were recorded at ambient temperature in deuterated solvents (CDCl_3_ or DMSO-*d*_6_) on a Bruker ADVANCE 400 spectrometer at 400.13 MHz or Bruker ADVANCE 500 spectrometer at 500 MHz. Chemical shifts are reported in parts per million (ppm) downfield from the tetramethylsilane reference (δ = 0) using the residual protonated solvent as an internal standard [^1^H: δ (CDCl_3_) = 7.26 ppm, δ (DMSO-*d*_6_) = 2.50 ppm]. Data for ^1^H NMR are given as follows: chemical shift {multiplicity, coupling constants [*J*, given in hertz (Hz)], integration and assignment}. Multiplicities in the ^1^H NMR spectra are quoted as follows: s = singlet, d = doublet, t = triplet, q = quartet, m = multiplet, dd = double doublet, and ddd = double double doublet. Splitting patterns that could not be interpreted or easily visualized were recorded as multiplets (m) or broad peaks (br).

^13^C NMR spectra were recorded at an ambient temperature in deuterated solvents (CDCl_3_ or DMSO-*d*_6_) on a Bruker ADVANCE 400 spectrometer at 100.61 MHz or Bruker ADVANCE 500 spectrometer at 125 MHz. Chemical shifts were measured in ppm relative to tetramethylsilane (δ = 0) using the following internal references: δ (CDCl_3_) = 77.0 ppm, δ (DMSO-*d*_6_) = 39.4 ppm.

##### High-Resolution Mass Spectrometry

High-resolution mass spectrometry (HRMS) spectra were recorded on a Micromass Q-TOF Premier Tandem Mass Spectrometer coupled to an HPLC instrument using electrospray ionization (ESI) mass spectrometry. Calibration was performed with an internal standard—positive mode: reserpine which gives *m*/*z* [M + H]^+^ = 609.2812, negative mode: taurocholic acid which gives *m*/*z* [M – H]^−^ = 514.2839.

##### Analytical TLC

Analytical TLC was performed on pre-coated Merck glass backed silica gel plates (Silica gel 60 F_254_) and visualized by exposure to ultraviolet light (254 nm) and/or staining with an appropriate reagent followed by heating if required.

##### Flash Column Chromatography

Flash column chromatography was carried out on Merck Kieselgel 60 (40–63 μm) under a positive pressure of N_2_ gas.

##### Liquid Chromatography–Mass Spectrometry

Liquid chromatography–mass spectrometry (LC–MS) spectra were recorded using a Shimadzu LCMS-2020 equipped with an XTerra MS C18 column (4.6 × 50 mm, 2.5 μm) and a flow rate of 0.6 mL/min. The eluent system consisted of eluent A (H_2_O with 0.1% formic acid, HPLC grade) and eluent B (MeCN with 0.1% formic acid, HPLC grade) with the following conditions: 0.0–2.5 min 90% A/10% B, then a linear gradient from 2.5 to 5.5 min to a final composition of 5% A/95% B that was maintained for a further 2.5 min, and then adjusted to 10% A/90% B over 10.5 min and held for 1.5 min. Total run time = 12 min.

##### Analytical Reverse-phase HPLC

Analytical reverse-phase HPLC was carried out on an XSELECT CSH C18 column 50 × 6 mm (particle size: 2.5 μm) at a flow rate of 1.0 mL/min. The eluent system consisted of eluent A (H_2_O with 0.1% TFA, HPLC grade) and eluent B (MeCN with 0.1% TFA, HPLC grade) with the following gradient conditions: initial fixed composition 5% B to 50% B over 20 min, then increased to 95% B over 2 min, held for 2 min at 95% B, and then returned to 5% B in 1 min. Total duration of the gradient run was 25 min.

All compounds that underwent biological testing were >95% pure by HPLC analysis.

##### *N*,*N*′-(2-Methoxy-1,4-phenylene)bis(4-methoxybenzenesulfonamide) **19**

To a solution of **13** (1.63 g, 9.7 mmol) in EtOH/H_2_O (220 mL, 4:1 v/v) was added AcOH (9.0 mL) and the mixture was brought to reflux. Fe (2.7 g, 48.25 mmol) was then added portion-wise and stirring was continued for 1 h. On completion, the reaction was left to cool to rt, diluted with H_2_O (50 mL) and basified to pH 8–9 with sat. NaHCO_3_. The reaction mixture was filtered through Celite and the filtrate was extracted with EtOAc (3 × 80 mL). The combined organic layers were washed with H_2_O (3 × 150 mL), sat. brine (3 × 150 mL), dried over anhydrous Na_2_SO_4_, and evaporated to dryness under reduced pressure. The crude product was sequentially dissolved in DCM (20 mL) and a solution of 4-methoxybenzenesulfonyl chloride (4.21 g, 20.3 mmol) in pyridine (2.4 mL, 29.10 mmol) was added to the reaction mixture. The reaction was stirred at rt overnight and following the addition of a small volume of hexane to aid compound precipitation, the crude solid was collected by filtration and washed with Et_2_O. Flash chromatography (EtOAc/hexane 7:3 v/v) afforded **19** (962 mg, 35%) as a white powder. ^1^H NMR (500 MHz, DMSO): δ 10.09 (s, 1H), 9.16 (s, 1H), 7.66 (d, *J* = 8.9 Hz, 2H), 7.58–7.41 (m, 2H), 7.06 (d, *J* = 8.9 Hz, 2H), 7.01 (d, *J* = 8.5 Hz, 1H), 6.98 (d, *J* = 8.9 Hz, 2H), 6.63–6.50 (m, 2H), 3.81 (s, 6H). ^13^C NMR (126 MHz, DMSO): δ 162.4, 162.1, 153.0, 136.6, 132.1, 130.0, 128.9, 128.7, 126.4, 121.2, 114.2, 113.7, 111.5, 103.5, 55.6, 55.5, 55.2.

##### *N*,*N*′-(2-Ethoxy-1,4-phenylene)bis(4-methoxybenzenesulfonamide) **20**

Prepared analogously to compound **19** from **14** (0.644 g, 3.54 mmol) and 4-methoxybenzenesulfonyl chloride (1.5 g, 7.44 mmol). Flash chromatography (EtOAc/hexane 7:3 v/v) afforded **20** (854 mg, 49%) as a white powder. ^1^H NMR (500 MHz, DMSO-*d*_6_): δ (ppm) 10.04 (s, 1H), 9.06 (s, 1H), 7.65 (d, *J* = 8.9 Hz, 2H), 7.48 (d, *J* = 8.9 Hz, 2H), 7.18–7.05 (m, 3H), 6.96 (d, *J* = 8.9 Hz, 2H), 6.63–6.44 (m, 2H), 3.76 (s, 3H), 3.74 (s, 3H), 3.56 (q, *J* = 6.9 Hz, 1H), 1.04 (t, *J* = 6.9 Hz, 1H). ^13^C NMR (125 MHz, DMSO-*d*_6_): δ (ppm) 162.4, 162.1, 152.1, 136.6, 132.1, 130.7, 128.9, 128.6, 126.4, 121.2, 114.1, 113.7, 111.3, 104.1, 63.0, 55.3, 55.2, 13.8.

##### *N*,*N*′-(2-Butoxy-1,4-phenylene)bis(4-methoxybenzenesulfonamide) **21**

Prepared analogously to compound **19** from **15** (0.609 g, 2.90 mmol) and 4-methoxybenzenesulfonyl chloride (1.3 g, 6.38 mmol). Flash chromatography (DCM 100%) afforded **21** (952 mg, 63%) as a white powder. ^1^H NMR (500 MHz, DMSO-*d*_6_): δ (ppm) 7.64 (d, *J* = 8.9 Hz, 2H), 7.45 (d, *J* = 8.9 Hz, 2H), 7.05 (dd, *J* = 8.7, 7.0 Hz, 3H), 6.95 (d, *J* = 8.9 Hz, 2H), 6.59–6.51 (m, 2H), 3.79 (s, 3H), 3.75 (s, 3H), 3.49 (t, *J* = 6.6 Hz, 2H), 1.42–1.10 (m, 4H), 0.83 (t, *J* = 7.4 Hz, 3H). ^13^C NMR (125 MHz, DMSO-*d*_6_): δ (ppm) 162.4, 162.2, 152.3, 136.6, 132.6, 130.9, 129.0, 128.6, 126.7, 121.2, 114.3, 113.8, 111.3, 104.6, 67.1, 55.6, 55.1, 30.1, 18.8, 13.7.

##### *N*,*N*′-[2-(Benzyloxy)-1,4-phenylene]bis(4-methoxybenzenesulfonamide) **22**

Prepared analogously to compound **19** from **16** (0.498 g, 2.04 mmol) and 4-methoxybenzenesulfonyl chloride (885 mg, 4.28 mmol). Flash chromatography (EtOAc/hexane 1:1 v/v) afforded **22** (656 mg, 58%) as a white powder. ^1^H NMR (500 MHz, DMSO-*d*_6_): δ (ppm) 10.06 (s, 1H), 9.22 (s, 1H), 7.54 (d, *J* = 8.9 Hz, 2H), 7.47 (d, *J* = 8.9 Hz, 2H), 7.35–7.28 (m, 3H), 7.25–7.17 (m, 2H), 7.05 (d, *J* = 8.5 Hz, 1H), 6.99 (d, *J* = 8.9 Hz, 2H), 6.86 (d, *J* = 8.9 Hz, 2H), 6.67 (d, *J* = 2.2 Hz, 1H), 6.53 (dd, *J* = 8.6, 2.2 Hz, 1H), 4.75 (s, 2H), 3.79 (s, 3H), 3.76 (s, 3H). ^13^C NMR (125 MHz, DMSO-*d*_6_): δ (ppm) 162.3, 162.1, 151.9, 136.6, 136.3, 132.2, 130.8, 128.9, 128.6, 128.5, 127.3, 126.9, 126.8, 121.2, 114.2, 113.8, 111.6, 104.5, 69.2, 55.6, 55.5.

##### *N*,*N*′-(2-Phenoxy-1,4-phenylene)bis(4-methoxybenzenesulfonamide) **23**

Prepared analogously to compound **19** from **17** (0.600 g, 2.61 mmol) and 4-methoxybenzenesulfonyl chloride (1.1 g, 5.48 mmol). Recrystallization from acetone/H_2_O afforded **23** (615 mg, 44%) as white crystals. ^1^H NMR (500 MHz, DMSO-*d*_6_): δ (ppm) 10.03 (s, 1H), 9.57 (s, 1H), 7.53–7.45 (m, 4H), 7.30–7.24 (m, 2H), 7.20 (d, *J* = 8.7 Hz, 1H), 7.12 (t, *J* = 7.4 Hz, 1H), 7.07–7.01 (m, 2H), 6.93–6.87 (m, 2H), 6.76 (dd, *J* = 8.7, 2.4 Hz, 1H), 6.45–6.40 (m, 2H), 6.36 (d, *J* = 2.4 Hz, 1H), 3.83 (s, 3H), 3.78 (s, 3H). ^13^C NMR (125 MHz, DMSO-*d*_6_): δ (ppm) 162.5, 162.3, 155.2, 150.5, 136.3, 131.1, 130.4, 129.6, 128.0.

##### *N*,*N*′-([1,1′-Biphenyl]-2,5-diyl)bis(4-methoxybenzenesulfonamide) **24**

Prepared analogously to compound **19** from **18** (1.300 g, 5.84 mmol) and 4-methoxybenzenesulfonyl chloride (2.53 g, 12.26 mmol). Flash chromatography (DCM 100%) afforded **24** (2.3 g, 75%) as an off-white fluffy powder. ^1^H NMR (500 MHz, DMSO-*d*_6_): δ (ppm) 10.26 (s, 1H), 9.20 (s, 1H), 7.74–7.65 (m, 2H), 7.37–7.28 (m, 5H), 7.11–7.03 (m, 4H), 7.01 (dd, *J* = 8.7, 2.6 Hz, 1H), 6.95–6.89 (m, 4H), 3.83 (s, 3H), 3.82 (s, 3H). ^13^C NMR (125 MHz, DMSO-*d*_6_): δ (ppm) 162.5, 162.1, 139.9, 138.1, 136.4, 132.1, 130.8, 128.9, 128.8, 128.7, 128.4, 127.9, 127.2, 121.8, 119.1, 114.4, 114.0, 55.6, 55.5. LC–MS *m*/*z* (ESI): 523.00 [M – H]^−^, *t*_R_ = 6.50 min, purity: >95%.

##### Diethyl 2,2′-((2-Methoxy-1,4-phenylene)bis(((4-methoxyphenyl)sulfonyl)azanediyl))diacetate **25**

To a solution of **19** (847 mg, 1.77 mmol) in DMF (4 mL) were added K_2_CO_3_ (739 mg, 5.31 mmol) and ethyl bromoacetate (490 μL, 4.42 mmol), and the reaction mixture was stirred at rt for 4 h. The reaction was quenched with H_2_O (30 mL) and acidified with 1 N HCl to pH 5. The resulting precipitate was collected by filtration, washed with H_2_O, and dried in a vacuum desiccator overnight. Flash chromatography (EtOAc/hexane 6:4 v/v) afforded **25** (765 mg, 66%) as a white solid. ^1^H NMR (500 MHz, DMSO): δ 7.62 (d, *J* = 8.9 Hz, 2H), 7.52 (d, *J* = 8.9 Hz, 2H), 7.30 (d, *J* = 8.5 Hz, 1H), 7.10 (d, *J* = 8.9 Hz, 2H), 7.06 (d, *J* = 8.9 Hz, 2H), 6.80 (dd, *J* = 8.5, 2.2 Hz, 1H), 6.62 (d, *J* = 2.2 Hz, 1H), 4.52 (s, 2H), 4.30 (s, 2H), 4.13–4.00 (m, 4H), 3.21 (s, 3H), 1.20–1.07 (m, 6H). ^13^C NMR (126 MHz, DMSO): δ 168.8, 168.6, 162.9, 162.6, 155.4, 141.0, 132.9, 130.9, 129.7, 129.6, 129.3, 125.4, 119.2, 114.3, 113.9, 111.2, 60.9, 60.7, 55.7, 55.6, 55.2, 51.9, 50.8, 13.9.

##### Diethyl 2,2′-((2-Ethoxy-1,4-phenylene)bis(((4-methoxyphenyl)sulfonyl)azanediyl))diacetate **26**

Prepared analogously to compound **25** from **20** (500 mg, 1.02 mmol) and ethyl bromoacetate (280 μL, 2.55 mmol). Flash chromatography (EtOAc/hexane 1:1 v/v) afforded **26** (523 mg, 77%) as a white powder. ^1^H NMR (500 MHz, DMSO-*d*_6_): δ (ppm) 7.61 (d, *J* = 8.9 Hz, 1H), 7.47 (d, *J* = 8.9 Hz, 1H), 7.32 (d, *J* = 8.5 Hz, 1H), 7.11–7.01 (m, 2H), 6.79 (dd, *J* = 8.5, 2.3 Hz, 1H), 6.59 (d, *J* = 2.2 Hz, 1H), 4.52–4.48 (m, 2H), 4.29–4.23 (m, 2H), 4.16–4.02 (m, 4H), 3.84 (s, 3H), 3.81 (s, 3H), 3.46 (q, *J* = 6.9 Hz, 2H), 1.14 (t, *J* = 7.1 Hz, 6H), 0.86 (t, *J* = 6.9 Hz, 3H). ^13^C NMR (125 MHz, DMSO-*d*_6_): δ (ppm) 168.9, 168.6, 162.8, 162.6, 154.6, 140.5, 133.2, 131.2, 129.3, 129.2, 129.0, 125.9, 118.3, 114.3, 113.8, 111.7, 63.2, 60.2, 60.1, 55.6, 55.4, 51.4, 50.5, 13.9, 13.1.

##### Diethyl 2,2′-((2-Butoxy-1,4-phenylene)bis(((4-methoxyphenyl)sulfonyl)azanediyl))diacetate **27**

Prepared analogously to compound **25** from **21** (150 mg, 0.29 mmol) and ethyl bromoacetate (80 μL, 0.72 mmol). Flash chromatography (DCM 100%) afforded **27** (143 mg, 72%) as a white powder. ^1^H NMR (500 MHz, DMSO-*d*_6_): δ (ppm) 7.61 (d, *J* = 8.9 Hz, 2H), 7.47 (d, *J* = 8.9 Hz, 2H), 7.32 (d, *J* = 8.5 Hz, 1H), 7.08 (d, *J* = 9.0 Hz, 2H), 7.03 (d, *J* = 8.9 Hz, 2H), 6.79 (dd, *J* = 8.5, 2.2 Hz, 1H), 6.59 (d, *J* = 2.2 Hz, 1H), 4.50 (s, 2H), 4.27 (s, 2H), 4.07 (q, *J* = 7.1 Hz, 4H), 3.84 (s, 3H), 3.82 (s, 3H), 3.46 (q, *J* = 6.9 Hz, 2H), 1.18–1.09 (m, 6H), 0.86 (t, *J* = 6.9 Hz, 3H). ^13^C NMR (125 MHz, DMSO-*d*_6_): δ (ppm) 168.9, 168.8, 162.8, 162.7, 154.5, 140.9, 133.6, 131.1, 129.7, 129.2, 125.3, 118.9, 114.3, 114.0, 111.7, 63.3, 60.8, 60.7, 55.8, 55.7, 51.9, 50.6, 13.7, 13.5.

##### Diethyl 2,2′-((2-(Benzyloxy)-1,4-phenylene)bis(((4-methoxyphenyl)sulfonyl)azanediyl))diacetate **28**

Prepared analogously to compound **25** from **22** (110 mg, 0.20 mmol) and ethyl bromoacetate (55 μL, 0.51 mmol). Flash chromatography (EtOAc/hexane 4:6 v/v) afforded **28** (121 mg, 83%) as a white solid. ^1^H NMR (500 MHz, DMSO-*d*_6_): δ (ppm) 7.62 (d, *J* = 8.9 Hz, 2H), 7.44 (d, *J* = 8.9 Hz, 2H), 7.37–7.30 (m, 4H), 7.12–7.02 (m, 4H), 6.89–6.76 (m, 4H), 4.67–4.58 (m, 2H), 4.54–4.48 (m, 2H), 4.32–4.27 (m, 2H), 4.13–4.01 (m, 4H), 3.84 (s, 3H), 3.75 (s, 3H), 1.20–1.07 (m, 6H). ^13^C NMR (125 MHz, DMSO-*d*_6_): δ (ppm) 168.9, 168.8, 162.9, 162.2, 154.6, 140.1, 135.4, 133.7, 130.8, 129.3, 129.1, 128.2, 127.2, 126.7, 125.8, 119.8, 114.7, 113.9, 112.1, 69.5, 60.9, 60.7, 55.7, 55.2, 51.3, 50.6, 13.9.

##### Diethyl 2,2′-((2-Phenoxy-1,4-phenylene)bis(((4-methoxyphenyl)sulfonyl)azanediyl))diacetate **29**

Prepared analogously to compound **25** from **23** (450 mg, 0.83 mmol) and ethyl bromoacetate (180 μL, 1.62 mmol). Recrystallization from EtOAc afforded **29** (450 mg, 76%) as white crystals. ^1^H NMR (500 MHz, DMSO-*d*_6_): δ (ppm) 7.59 (d, *J* = 8.9 Hz, 2H), 7.53–7.44 (m, 3H), 7.31 (t, *J* = 7.9 Hz, 2H), 7.18 (t, *J* = 7.4 Hz, 1H), 7.06–6.95 (m, 5H), 6.57 (d, *J* = 7.7 Hz, 2H), 6.36 (d, *J* = 2.4 Hz, 1H), 4.45–4.39 (m, 4H), 4.07–4.00 (m, 4H), 3.87 (s, 3H), 3.80 (s, 3H), 1.20–1.13 (m, 6H). ^13^C NMR (125 MHz, DMSO-*d*_6_): δ (ppm) 168.7, 168.4, 162.7, 162.6, 153.9, 153.6, 140.6, 133.5, 130.8, 129.7, 129.6, 129.4, 129.3, 126.7, 124.5, 120.9, 119.3, 115.0, 114.3, 114.1, 60.9, 60.7, 55.7, 55.6, 51.7, 51.2, 13.9, 13.8.

##### Diethyl 2,2′-([1,1′-Biphenyl]-2,5-diylbis(((4-methoxyphenyl)sulfonyl)azanediyl))diacetate **30**

Prepared analogously to compound **25** from **24** (500 mg, 0.95 mmol) and ethyl bromoacetate (260 μL, 2.38 mmol). Flash chromatography (EtOAc/hexane 4:6 v/v) afforded 30 (580 mg, 87%) as a white solid. ^1^H NMR (500 MHz, DMSO-*d*_6_): δ (ppm) 7.62 (d, *J* = 8.9 Hz, 2H), 7.52 (d, *J* = 8.9 Hz, 2H), 7.39–7.33 (m, 3H), 7.28–7.16 (m, 4H), 7.12–7.00 (m, 5H), 4.53 (s, 2H), 4.08 (q, *J* = 7.1 Hz, 2H), 3.91 (q, *J* = 7.1 Hz, 2H), 3.87 (s, 3H), 3.84 (s, 3H), 1.12 (t, *J* = 7.1 Hz, 3H), 1.02 (t, *J* = 7.1 Hz, 3H). ^13^C NMR (125 MHz, DMSO-*d*_6_): δ (ppm) 168.6, 168.1, 162.9, 162.8, 141.3, 139.5, 137.7, 135.9, 130.7, 130.6, 129.8, 129.7, 129.6, 129.5, 128.5, 128.1, 127.7, 126.8, 114.4, 114.2, 60.9, 60.7, 55.7, 51.9, 51.8, 13.9, 13.7. LC–MS *m*/*z* (ESI): 698.15 [M + H]^+^, *t*_R_ = 7.47 min, purity: >95%.

##### 2,2′-((2-Methoxy-1,4-phenylene)bis(((4-methoxyphenyl)sulfonyl)azanediyl))diacetic Acid **10**

To a solution of **25** (505 mg, 0.78 mmol) in THF/MeOH (10 mL, 1:1 v/v) was added 1 N NaOH (5 mL) and the reaction was stirred at rt for 2 h. On completion, the reaction was acidified with 1 N HCl to pH 1–2 and the resulting precipitate was collected by filtration, washed thoroughly with H_2_O, and dried in a vacuum desiccator overnight to afford **10** (142 mg, 29%) as a white solid. ^1^H NMR (500 MHz, DMSO): δ 12.82 (br, 2H), 7.61 (d, *J* = 8.9 Hz, 2H), 7.49 (d, *J* = 8.9 Hz, 2H), 7.32 (d, *J* = 8.5 Hz, 1H), 7.10 (d, *J* = 8.9 Hz, 2H), 7.05 (d, *J* = 8.9 Hz, 2H), 6.81 (dd, *J* = 8.5, 2.2 Hz, 1H), 6.60 (d, *J* = 2.2 Hz, 1H), 4.41 (s, 2H), 4.20 (s, 2H), 3.85 (s, 6H), 3.18 (s, 3H). ^13^C NMR (126 MHz, DMSO): δ 170.3, 170.0., 162.8, 162.5, 155.3, 141.0, 133.1, 131.2, 129.8, 129.6, 129.2, 125.4, 119.1, 114.3, 113.9, 111.0, 55.7, 55.7, 55.2, 51.8, 50.7.^14,24^

##### 2,2′-((2-Ethoxy-1,4-phenylene)bis(((4-methoxyphenyl)sulfonyl)azanediyl))diacetic Acid **31**

Prepared analogously to compound **10** from **26** (200 mg, 0.30 mmol). Flash chromatography (DCM/EtOAc/formic acid 90:9:1 v/v/v) afforded 31 (149 mg, 82%) as a white solid. ^1^H NMR (500 MHz, DMSO-*d*_6_): δ (ppm) 13.32–12.22 (br, 2H), 7.61 (dd, *J* = 8.9 Hz, 2H), 7.46 (t, *J* = 8.9 Hz, 2H), 7.35 (d, *J* = 8.5 Hz, 1H), 7.19–7.02 (m, 4H), 6.80 (dd, *J* = 8.5, 2.3 Hz, 1H), 6.59 (d, *J* = 2.3 Hz, 1H), 4.51–4.36 (m, 2H), 4.31–4.08 (m, 2H), 3.86 (s, 3H), 3.84 (s, 3H), 3.45 (q, *J* = 6.9 Hz, 2H), 0.87 (t, *J* = 6.9 Hz, 3H). ^13^C NMR (125 MHz, DMSO-*d*_6_): δ (ppm) 170.7, 170.0, 162.7, 162.5, 154.9, 140.1, 133.8, 131.6, 129.8, 129.4, 129.3, 125.2, 118.8, 114.1, 113.9, 111.4, 63.2, 55.7, 55.6, 51.8, 50.5, 30.6, 22.7, 13.7, 13.6. LC–MS *m*/*z* (ESI): 1214.05 [2M – H]^−^, *t*_R_ = 5.76 min, purity: >95%. HRMS (ESI): calcd for C_26_H_28_N_2_O_11_S_2_ [M – H]^−^, 607.1057; found, 607.1032. HPLC: *t*_R_ = 8.44 min, purity: >95%.

##### 2,2′-((2-Butoxy-1,4-phenylene)bis(((4-methoxyphenyl)sulfonyl)azanediyl))diacetic Acid **32**

Prepared analogously to compound **10** from **27** (60 mg, 0.09 mmol). Flash chromatography (DCM/EtOAc/formic acid 80:19:1 v/v/v) afforded **32** (40 mg, 72%) as a white solid. ^1^H NMR (400 MHz, DMSO-*d*_6_): δ (ppm) 13.32–12.75 (br, 2H), 7.61 (d, *J* = 8.9 Hz, 2H), 7.46 (d, *J* = 8.9 Hz, 2H), 7.35 (d, *J* = 8.5 Hz, 1H), 7.12–7.01 (m, 4H), 6.82 (dd, *J* = 8.5, 2.2 Hz, 1H), 6.56 (d, *J* = 2.2 Hz, 1H), 4.40 (s, 2H), 4.16 (s, 2H), 3.86 (s, 3H), 3.84 (s, 3H), 3.43–3.32 (m, 2H), 1.23–1.01 (m, 4H), 0.86–0.68 (m, 3H). ^13^C NMR (100 MHz, DMSO-*d*_6_): δ (ppm) 170.2, 169.9, 162.7, 162.3, 154.1, 140.8, 133.6, 131.5, 129.4, 129.3, 129.2, 125.2, 119.1, 114.2, 113.9, 111.5, 111.4, 55.7, 55.6, 29.9, 18.3, 13.4. LC–MS *m*/*z* (ESI): 635.00 [M–H]^−^, *t*_R_ = 6.19 min, purity: >95%. HRMS (ESI): calcd for C_28_H_32_N_2_O_11_S_2_ [M – H]^−^, 635.1369; found, 635.1350. HPLC: *t*_R_ = 9.28 min, purity: >95%.

##### 2,2′-((2-(Benzyloxy)-1,4-phenylene)bis(((4-methoxyphenyl)sulfonyl)azanediyl))diacetic Acid **11**

Prepared analogously to compound **10** from **28** (100 mg, 0.15 mmol). The crude product was re-suspended in EtOAc and filtered to afford **11** (69 mg, 69%) as a white solid. ^1^H NMR (500 MHz, DMSO-*d*_6_): δ (ppm) 13.07–12.30 (br, 2H), 7.61 (d, *J* = 8.9 Hz, 2H), 7.42 (d, *J* = 8.9 Hz, 2H), 7.37 (d, *J* = 8.5 Hz, 1H), 7.35–7.28 (m, 3H), 7.08 (d, *J* = 9.0 Hz, 2H), 7.07–7.01 (m, 2H), 6.87–6.81 (m, 3H), 6.77 (d, *J* = 2.2 Hz, 1H), 4.59 (s, 2H), 4.41 (s, 2H), 4.21 (s, 2H), 3.84 (s, 3H), 3.74 (s, 3H). ^13^C NMR (125 MHz, DMSO-*d*_6_): δ (ppm) 170.3, 170.0, 162.8, 162.4, 154.4, 140.9, 135.8, 133.3, 131.2, 129.8, 129.7, 129.0, 128.2, 127.7, 126.8, 125.6, 119.3, 114.3, 113.9, 111.9, 69.5, 55.7, 55.5, 51.8, 50.5. LC–MS *m*/*z* (ESI): 669.20 [M – H]^−^, *t*_R_ = 6.17 min, purity: >95%. HRMS (ESI): calcd for C_31_H_30_N_2_O_11_S_2_ [M – H]^−^, 669.1213; found, 669.1183. HPLC: *t*_R_ = 9.46 min, purity: >95%.

##### 2,2′-((2-Phenoxy-1,4-phenylene)bis(((4-methoxyphenyl)sulfonyl)azanediyl))diacetic Acid **33**

Prepared analogously to compound **10** from **29** (190 mg, 0.83 mmol). The crude product was recrystallized from MeOH/H_2_O to afford **33** (130 mg, 56%) as white crystals. ^1^H NMR (500 MHz, DMSO-*d*_6_): δ (ppm) 12.92–12.75 (br, 2H), 7.60–7.52 (m, 2H), 7.51–7.44 (m, 3H), 7.37–7.23 (m, 2H), 7.15 (dd, *J* = 7.4, 7.3 Hz, 1H), 7.07–6.89 (m, 5H), 6.54 (dd, *J* = 10.1, 9.1 Hz, 2H), 6.35 (d, *J* = 6.4 Hz, 1H), 4.32–4.29 (m, 4H), 3.86 (s, 3H), 3.79 (s, 3H). ^13^C NMR (125 MHz, DMSO-*d*_6_): δ (ppm) 170.2, 169.2, 162.7, 162.4, 154.0, 153.2, 140.0, 133.7, 131.0, 129.7, 129.5, 129.3, 129.2, 126.2, 124.4, 120.1, 119.2, 115.0, 114.3, 114.1, 55.7, 55.6, 51.6, 51.2. LC–MS *m*/*z* (ESI): 1310.65 [2M – H]^−^, *t*_R_ = 6.10 min, purity: >95%. HRMS (ESI): calcd for C_30_H_28_N_2_O_11_S_2_ [M + H]^+^, 655.1057; found, 655.1057. HPLC: *t*_R_ = 9.20 min, purity: >95%.

##### 2,2′-([1,1′-Biphenyl]-2,5-diylbis(((4-methoxyphenyl)sulfonyl)azanediyl))diacetic Acid **34**

Prepared analogously to compound **10** from **30** (170 mg, 0.24 mmol). The crude product was purified by flash chromatography (CHCl_3_/EtOAc/formic acid 80:19:1 v/v/v) to afford **34** (105 mg, 68%) as a white solid. ^1^H NMR (400 MHz, DMSO-*d*_6_): δ (ppm) 13.15–12.43 (br, 2H), 7.61 (d, *J* = 8.9 Hz, 2H), 7.53 (d, *J* = 8.9 Hz, 2H), 7.38–7.28 (m, 4H), 7.25–7.17 (m, 3H), 7.09 (d, *J* = 9.0 Hz, 2H), 7.04 (d, *J* = 9.0 Hz, 2H), 6.99 (d, *J* = 2.6 Hz, 1H), 4.43 (s, 2H), 3.87 (s, 3H), 3.85 (s, 3H). ^13^C NMR (100 MHz, DMSO-*d*_6_): δ (ppm) 169.9, 169.4, 162.8, 162.6, 141.2, 139.9, 137.3, 135.8, 131.3, 130.7, 129.8, 129.6, 129.5, 128.4, 128.1, 127.6, 126.4, 114.4, 114.2, 55.3, 55.1, 51.5. HRMS (ESI): calcd for C_30_H_28_N_2_O_10_S_2_ [M + H]^+^, 641.1263; found, 641.1260. LC–MS *m*/*z* (ESI): 693.05 [M – H]^−^, *t*_R_ = 6.50 min, purity: >95%. HPLC: *t*_R_ = 9.17 min, purity: >95%.

##### Diethyl 2,2′-((2-Hydroxy-1,4-phenylene)bis(((4-methoxyphenyl)sulfonyl)azanediyl))diacetate **35**

To a solution of **28** (2.9 g, 4.36 mmol) and Pd/C (5% w/w, 600 mg) in EtOAc/EtOH (50 mL, 9:1 v/v) in a sealed flask was added TES (7.0 mL, 46.63 mmol) dropwise over a period of 1 h and stirring was continued for an additional 1 h. The reaction mixture was filtered through Celite, diluted with H_2_O (100 mL), and extracted with EtOAc (3 × 100 mL). The combined organic layers were washed with H_2_O (3 × 150 mL) and sat. brine (3 × 150 mL), dried over anhydrous MgSO_4_, and evaporated under reduced pressure to afford the phenol **35** (1.7 g, 61%) as a white solid. ^1^H NMR (400 MHz, CDCl_3_): δ (ppm) 8.26 (s, 1H), 7.70–7.62 (m, 2H), 7.62–7.54 (m, 2H), 6.97–6.87 (m, 4H), 6.77 (dd, *J* = 8.6, 2.4 Hz, 1H), 6.73–6.67 (m, 2H), 4.34 (s, 2H), 4.27 (q, *J* = 7.2 Hz, 2H), 4.16 (q, *J* = 7.1 Hz, 2H), 3.88 (s, 3H), 3.87 (s, 3H), 1.30 (t, *J* = 7.2 Hz, 3H), 1.24 (t, *J* = 7.2 Hz, 3H). ^13^C NMR (100 MHz, CDCl_3_): δ (ppm) 172.2, 168.8, 163.6, 163.5, 156.5, 142.6, 130.4, 130.3, 130.2, 130.1, 129.6, 125.1, 120.0, 116.1, 114.8, 114.6, 62.6, 61.8, 55.6, 55.5, 53.2, 52.2, 14.7, 14.6.

##### 2,2′-((2-Propoxy-1,4-phenylene)bis(((4-methoxyphenyl)sulfonyl)azanediyl))diacetic Acid **36**

To a solution of **35** (400 mg, 0.63 mmol) and K_2_CO_3_ (287 mg, 2.08 mmol) in acetone (10 mL) was added 1-iodopropane (100 μL, 1.04 mmol) and the resulting suspension was stirred at reflux for 16 h. On completion, the reaction mixture was cooled to rt, diluted with H_2_O (80 mL), and extracted with EtOAc (3 × 100 mL). The combined organic layers were washed with H_2_O (3 × 200 mL) and sat. brine (3 × 200 mL), dried over anhydrous MgSO_4_, and evaporated to dryness under reduced pressure. The crude product was dissolved in THF/MeOH (10 mL, 1:1 v/v) and a solution of 1 N NaOH (6.3 mL) was added. The reaction was stirred at 40 °C for 2 h and was then cooled to rt. The reaction mixture was acidified with 1 N HCl to pH 1–2 and the resulting precipitate was filtered, washed with H_2_O, DCM, and EtOAc, and dried in vacuo to afford **36** (160 mg, 42%) as a white solid. ^1^H NMR (500 MHz, DMSO-*d*_6_): δ (ppm) 13.15–12.66 (br, 2H), 7.61 (d, *J* = 8.9 Hz, 2H), 7.46 (d, *J* = 8.9 Hz, 2H), 7.35 (d, *J* = 8.5 Hz, 1H), 7.09 (d, *J* = 8.9 Hz, 2H), 7.04 (d, *J* = 8.9 Hz, 2H), 6.81 (dd, *J* = 8.5, 2.2 Hz, 1H), 6.57 (d, *J* = 2.2 Hz, 1H), 4.41 (s, 2H), 4.19 (s, 2H), 3.85 (s, 3H), 3.84 (s, 3H), 1.27–1.15 (m, 2H), 0.72 (t, *J* = 7.4 Hz, 3H). ^13^C NMR (125 MHz, DMSO-*d*_6_): δ (ppm) 170.3, 170.0, 162.8, 162.5, 154.5, 140.8, 133.3, 131.3, 129.8, 129.6, 129.0, 125.2, 119.0, 114.2, 113.9, 111.5, 69.0, 55.7, 55.6, 51.8, 50.4, 21.3, 10.0. LC–MS *m*/*z* (ESI): 1242.00 [2M – H]^−^, *t*_R_ = 5.96 min, purity: >95%. HRMS (ESI): calcd for C_27_H_30_N_2_O_11_S_2_ [M – H]^−^, 621.1213; found, 621.1186. HPLC: *t*_R_ = 9.01 min, purity: >95%.

##### 2,2′-((2-Isopropoxy-1,4-phenylene)bis(((4-methoxyphenyl)sulfonyl)azanediyl))diacetic Acid **37**

Prepared analogously to compound **36** from **35** (400 mg, 0.63 mmol) and 2-bromopropane (63 μL, 0.67 mmol). The crude product was re-suspended in boiling EtOAc and filtered to afford **37** (193 mg, 49%) as a white solid. ^1^H NMR (500 MHz, DMSO-*d*_6_): δ (ppm) 13.03–12.59 (br, 2H), 7.59 (d, *J* = 8.8 Hz, 2H), 7.51 (d, *J* = 8.8 Hz, 2H), 7.36 (d, *J* = 8.5 Hz, 1H), 7.06 (dd, *J* = 16.3, 8.9 Hz, 4H), 6.78 (dd, *J* = 8.5, 2.0 Hz, 1H), 6.55 (d, *J* = 1.7 Hz, 1H), 4.42 (s, 2H), 4.19 (d, *J* = 16.1 Hz, 2H), 4.13 (dq, *J* = 11.6, 5.8 Hz, 1H), 3.84 (s, 6H), 0.80 (d, *J* = 5.8 Hz, 6H). ^13^C NMR (125 MHz, DMSO-*d*_6_): δ (ppm) 170.4, 170.0, 162.7, 162.5, 153.0, 140.5, 133.5, 131.5, 129.8, 129.4, 129.1, 125.4, 118.6, 114.3, 114.0, 112.0, 69.2, 55.6, 55.6, 51.8, 50.4, 20.8. LC–MS *m*/*z* (ESI): 1242.10 [2M – H]^−^, *t*_R_ = 5.88 min, purity: >95%. HRMS (ESI): calcd for C_27_H_30_N_2_O_11_S_2_ [M – H]^−^, 621.1213; found, 621.1202. HPLC: *t*_R_ = 9.32 min, purity: >95%.

##### 2,2′-((2-((3-Methoxybenzyl)oxy)-1,4-phenylene)bis(((4-methoxyphenyl)sulfonyl)azanediyl))diacetic Acid **38**

Prepared analogously to compound **36** from **35** (176 mg, 0.28 mmol) and 3-methoxybenzyl bromide (45 μL, 0.30 mmol). The crude product was re-suspended in boiling MeOH and filtered to afford **38** (29 mg, 15%) as a white solid. ^1^H NMR (500 MHz, DMSO-*d*_6_): δ (ppm) 12.96–12.59 (br, 2H), 7.65–7.57 (m, 2H), 7.46–7.39 (m, 2H), 7.34 (d, *J* = 8.5 Hz, 1H), 7.29–7.10 (m, 3H), 6.90–6.74 (m, 6H), 6.64 (d, *J* = 7.6 Hz, 1H), 4.59 (s, 2H), 4.41 (s, 2H), 4.23 (s, 2H), 3.85 (s, 3H), 3.77 (s, 3H), 3.75 (s, 3H). ^13^C NMR (125 MHz, DMSO-*d*_6_): δ (ppm) 170.3, 169.9, 162.8, 162.4, 159.2, 154.4, 140.9, 137.4, 133.2, 131.1, 129.8, 129.7, 129.3, 129.0, 125.6, 119.2, 118.8, 114.3, 113.9, 113.3, 112.2, 111.8, 69.3, 55.7, 55.4, 54.9, 51.8, 50.6. LC–MS *m*/*z* (ESI): 1399.10 [2M – H]^−^, *t*_R_ = 6.20 min, purity: >95%. HRMS (ESI): calcd for C_32_H_32_N_2_O_12_S_2_ [M – H]^−^, 699.1318; found, 699.1282. HPLC: *t*_R_ = 9.34 min, purity: >95%.

##### 2,2′-((2-Hydroxy-1,4-phenylene)bis(((4-methoxyphenyl)sulfonyl)azanediyl))diacetic Acid **39**

Prepared analogously to compound **10** from **35** (100 mg, 0.16 mmol). The crude product was re-suspended in boiling EtOAc and filtered to afford **39** (65 mg, 71%) as a white solid. ^1^H NMR (500 MHz, DMSO-*d*_6_): δ (ppm) 13.35–12.86 (br, 2H), 9.85 (s, 1H), 7.59 (d, *J* = 8.9 Hz, 2H), 7.53 (d, *J* = 8.9 Hz, 2H), 7.18–7.09 (m, 3H), 7.02 (d, *J* = 8.9 Hz, 2H), 6.67 (d, *J* = 2.4 Hz, 1H), 6.52 (dd, *J* = 8.6, 2.4 Hz, 1H), 4.32 (s, 2H), 4.23 (s, 2H), 3.85 (s, 3H), 3.83 (s, 3H). ^13^C NMR (125 MHz, DMSO-*d*_6_): δ (ppm) 170.9, 169.9, 162.7, 162.4, 154.2, 140.6, 132.5, 131.3, 130.0, 129.5, 129.3, 124.1, 116.6, 115.1, 114.3, 113.9, 55.7, 55.6, 51.8, 50.6. HRMS (ESI): calcd for C_24_H_24_N_2_O_11_S_2_ [M + H]^+^, 579.0743; found, 579.0715. HPLC: *t*_R_ = 8.12 min, purity: >95%.

##### 2,2′-((2-(Benzyloxy)-1,4-phenylene)bis(((4-methoxyphenyl)sulfonyl)azanediyl))diacetamide **40**

To a solution of **11** (150 mg, 0.24 mmol) in dry DMF (4 mL) were added successively dry pyridine (39 μL, 0.48 mmol), Boc_2_O (155 mg, 0.72 mmol), and (NH_4_)_2_CO_3_ (34 mg, 0.34 mmol), and the reaction mixture was stirred under Ar for 19 h at rt. On completion, H_2_O (10 mL) was added and the resulting precipitate was collected by filtration, washed with H_2_O and Et_2_O, and dried in vacuo to give **40** (110 mg, 74%) as a white solid. ^1^H NMR (400 MHz, DMSO-*d*_6_): δ (ppm) 7.60–7.55 (m, 2H), 7.47–7.40 (m, 3H), 7.39–7.31 (m, 4H), 7.32–7.26 (br, 1H), 7.26–7.19 (br, 1H), 7.17–7.06 (m, 5H), 6.91 (d, *J* = 2.3 Hz, 1H), 6.89–6.83 (m, 2H), 6.80 (dd, *J* = 8.5, 2.3 Hz, 1H), 4.65 (s, 2H), 4.17 (s, 2H), 4.05 (s, 2H), 3.84 (s, 3H), 3.75 (s, 3H). ^13^C NMR (100 MHz, DMSO-*d*_6_): δ (ppm) 169.6, 168.9, 162.3, 162.1, 154.4, 141.2, 135.1, 132.9, 130.4, 129.7, 129.2, 128.2, 127.7, 126.4, 126.1, 119.1, 114.2, 113.3, 112.5, 69.6, 55.7, 55.4, 52.8, 52.1. HRMS (ESI): calcd for C_31_H_32_N_4_O_9_S_2_ [M – H]^−^, 667.1533; found, 667.1513. HPLC: *t*_R_ = 8.37 min, purity: >95%.

##### *N*,*N*′-[2-(Benzyloxy)-1,4-phenylene]bis[*N*-(cyanomethyl)-4-methoxybenzenesulfonamide] **41**

Prepared analogously to compound **25** from **22** (400 mg, 0.72 mmol) and bromoacetonitrile (130 μL, 1.80 mmol). Flash chromatography (EtOAc/hexane 4:6 v/v) afforded **41** (270 mg, 59%) as an off-white solid. ^1^H NMR (400 MHz, CDCl_3_): δ (ppm) 7.61–7.49 (m, 4H), 7.41–7.31 (m, 4H), 7.18–7.09 (m, 2H), 7.01 (d, *J* = 2.3 Hz, 1H), 6.98–6.91 (m, 2H), 6.86–6.79 (m, 2H), 6.72 (dd, *J* = 8.4, 2.4 Hz, 1H), 4.79 (s, 2H), 4.62–4.51 (m, 4H), 3.87 (s, 3H), 3.84 (s, 3H). ^13^C NMR (100 MHz, CDCl_3_): δ (ppm) 164.1, 163.6, 155.8, 140.9, 135.0, 133.3, 130.3, 130.2, 129.8, 128.8, 128.6, 128.3, 127.6, 126.3, 119.4, 115.1, 114.9, 114.5, 114.3, 113.8, 70.9, 55.8, 55.7, 39.2, 38.2. LC–MS *m*/*z* (ESI): 634.65 [M – H]^−^, *t*_R_ = 7.11 min, purity: >95%. HPLC: *t*_R_ = 11.13 min, purity: >95%.

##### *N*-((1*H*-Tetrazol-5-yl)methyl)-*N*-(4-((*N*-((1*H*-tetrazol-5-yl)methyl)-4-methoxyphenyl)sulfonamido)-2-(benzyloxy)phenyl)-4-methoxybenzenesulfonamide **42**

To a solution of **41** (200 mg, 0.32 mmol) in DMF (5 mL) were added NaN_3_ (105 mg, 1.61 mmol) and NH_4_Cl (105 mg, 1.96 mmol), and the reaction was stirred at 100 °C for 16 h. On completion, the reaction mixture was diluted with H_2_O (40 mL), extracted with EtOAc (3 × 40 mL), and the combined organic layers were washed with H_2_O (3 × 100 mL), sat. brine (3 × 100 mL), dried over anhydrous MgSO_4_, and evaporated to dryness under reduced pressure. The crude product was purified by flash chromatography (DCM/EtOAc/formic acid 80:19:1 v/v) to afford **42** (140 mg, 64%) as a white solid. ^1^H NMR (500 MHz, DMSO-*d*_6_): δ (ppm) 7.45 (d, *J* = 8.9 Hz, 2H), 7.40–7.32 (m, 5H), 7.24 (d, *J* = 8.2 Hz, 1H), 7.11 (d, *J* = 8.9 Hz, 2H), 7.09–7.04 (m, 2H), 6.86 (d, *J* = 8.9 Hz, 2H), 6.80–6.75 (m, 2H), 5.12 (s, 2H), 5.04 (s, 2H), 4.53 (s, 2H), 3.86 (s, 3H), 3.77 (s, 3H). ^13^C NMR (125 MHz, DMSO-*d*_6_): δ (ppm) 163.1, 162.7, 154.7, 140.3, 135.5, 133.1, 129.8, 129.1, 128.2, 127.8, 126.9, 125.4, 120.1, 114.4, 114.1, 113.0, 69.8, 55.8, 55.5. LC–MS *m*/*z* (ESI): 717.70 [M – H]^−^, *t*_R_ = 5.68 min, purity: >95%. HRMS (ESI): calcd for C_31_H_30_N_10_O_7_S_2_ [M + H]^+^, 719.1819; found, 719.1812. HPLC: *t*_R_ = 9.30 min, purity: >95%.

##### Ethyl 2-{*N*-[2-(Benzyloxy)-4-nitrophenyl]-4-methoxyphenylsulfonamido}acetate **43**

To a solution of **16** (10.0 g, 29.04 mmol) in DCM (100 mL) was added 4-methoxybenzenesulfonyl chloride (6.6 g, 31.94 mmol), DMAP (355 mg, 2.90 mmol), and pyridine (5.2 mL, 63.89 mmol), and the reaction was stirred for 24 h at rt. On completion, the reaction was diluted with H_2_O (100 mL) and acidified to pH 2–3 with 1 N HCl. The organic layer was isolated and the aqueous layer was extracted with DCM (3 × 100 mL). The combined organic portions were washed with 1 N HCl (3 × 200 mL), H_2_O (3 × 200 mL), and sat. brine (3 × 200 mL), dried over anhydrous Na_2_SO_4_, and evaporated to dryness under reduced pressure. The crude product was triturated with Et_2_O and used in the next step without further purification.

To a solution of the crude product in DMF (10 mL) was added ethyl bromoacetate (4.0 mL, 36.29 mmol) and K_2_CO_3_ (6.0 g, 43.55 mmol), and the reaction was stirred for 8 h at rt. On completion, the reaction mixture was acidified with 1 N HCl to pH 4–5 and the resulting precipitate was collected by filtration, washed with H_2_O, Et_2_O, and MeOH, and dried in vacuo to afford the ester **43** (7.6 g, 52%) as an off-white powder. ^1^H NMR (500 MHz, DMSO-*d*_6_): δ (ppm) 7.96–7.86 (m, 2H), 7.71 (d, *J* = 8.6 Hz, 1H), 7.53 (d, *J* = 8.9 Hz, 2H), 7.37–7.30 (m, 3H), 7.16–7.07 (m, 2H), 6.90 (d, *J* = 8.9 Hz, 2H), 5.02 (s, 2H), 4.39 (s, 2H), 4.06 (q, *J* = 7.1 Hz, 2H), 3.75 (s, 3H), 1.15 (t, *J* = 7.1 Hz, 3H). ^13^C NMR (125 MHz, DMSO-*d*_6_): δ (ppm) 168.6, 162.7, 154.9, 147.7, 135.5, 134.0, 132.9, 130.6, 129.1, 128.2, 127.8, 127.0, 115.5, 114.2, 108.2, 70.2, 60.8, 55.5, 50.2, 13.9.

##### *N*-[2-(Benzyloxy)-4-nitrophenyl]-*N*-(cyanomethyl)-4-methoxybenzenesulfonamide **44**

Prepared analogously to compound **43** from **16** (7.5 g, 21.77 mmol) and bromoacetonitrile (1.9 mL, 27.28 mmol). The crude product was crystallized from EtOAc/hexane to afford **44** (4.2 g, 43%) as off-white crystals. ^1^H NMR (500 MHz, DMSO-*d*_6_): δ (ppm) 7.96–7.90 (m, 1H), 7.66–7.54 (m, 3H), 7.40–7.29 (m, 3H), 7.21 (dd, *J* = 6.5, 2.6 Hz, 2H), 6.93 (d, *J* = 9.0 Hz, 2H), 5.08 (s, 2H), 4.79 (s, 2H), 3.78 (s, 3H). ^13^C NMR (125 MHz, DMSO-*d*_6_): δ (ppm) 163.1, 155.3, 148.3, 135.3, 132.7, 132.0, 129.5, 129.4, 128.3, 127.9, 127.2, 116.5, 115.8, 114.4, 108.5, 70.5, 55.6, 38.1.

##### Ethyl 2-{*N*-[2-(Benzyloxy)-4-(4-methoxyphenylsulfonamido)phenyl]-4-methoxyphenylsulfonamido}acetate **45**

Prepared analogously to compound **19** from **43** (3.2 g, 6.39 mmol) and 4-methoxybenzenesulfonyl chloride (1.45 g, 7.03 mmol). Flash chromatography (DCM/Et_2_O 9:1 v/v) afforded **45** (2.8 g, 69%) as a white solid. ^1^H NMR (500 MHz, DMSO-*d*_6_): δ (ppm) 10.36 (s, 1H), 7.68 (d, *J* = 8.9 Hz, 2H), 7.41 (d, *J* = 8.9 Hz, 2H), 7.37–7.28 (m, 3H), 7.22 (d, *J* = 8.5 Hz, 1H), 7.12–7.02 (m, 4H), 6.85 (d, *J* = 8.9 Hz, 2H), 6.75 (d, *J* = 2.2 Hz, 1H), 6.64 (dd, *J* = 8.6, 2.2 Hz, 1H), 4.69 (s, 2H), 4.26 (s, 2H), 4.02 (q, *J* = 7.1 Hz, 2H), 3.80 (s, 3H), 3.75 (s, 3H), 1.09 (t, *J* = 7.1 Hz, 3H). ^13^C NMR (125 MHz, DMSO-*d*_6_): δ (ppm) 168.9, 162.5, 162.4, 154.9, 139.3, 136.0, 133.6, 131.3, 131.0, 129.5, 129.0, 128.2, 127.6, 126.7, 121.9, 114.4, 113.9, 110.3, 103.5, 69.2, 60.6, 55.6, 55.5, 50.8, 13.9.

##### *N*-{2-(Benzyloxy)-4-[(4-methoxyphenyl)sulfonamido]phenyl}-*N*-(cyanomethyl)-4-methoxybenzenesulfonamide **46**

Prepared analogously to compound **19** from **44** (3.8 g, 8.38 mmol) and 4-methoxybenzenesulfonyl chloride (1.9 g, 9.22 mmol). Flash chromatography (EtOAc/hexane 3:7 v/v) afforded **46** (2.3 g, 46%) as a white solid. ^1^H NMR (500 MHz, DMSO-*d*_6_): δ (ppm) 10.46 (s, 1H), 7.67 (dd, *J* = 6.9, 5.0 Hz, 2H), 7.48 (dd, *J* = 6.9, 5.0 Hz, 2H), 7.36–7.30 (m, 3H), 7.17 (dd, *J* = 6.4, 2.9 Hz, 2H), 7.12–6.98 (m, 3H), 6.89 (d, *J* = 9.0 Hz, 2H), 6.80 (d, *J* = 2.3 Hz, 1H), 6.66 (dd, *J* = 8.5, 2.3 Hz, 1H), 4.75 (s, 2H), 4.64 (s, 2H), 3.81 (s, 3H), 3.78 (s, 3H). ^13^C NMR (125 MHz, DMSO-*d*_6_): δ (ppm) 162.8, 162.5, 155.3, 140.1, 135.9, 132.3, 131.0, 129.9, 129.3, 128.9, 128.2, 127.7, 127.0, 120.9, 116.8, 114.4, 114.2, 110.5, 103.6, 69.4, 55.6, 55.5, 38.6.

##### Ethyl 2-(*N*-(2-(Benzyloxy)-4-(*N*-(cyanomethyl)-4-methoxyphenylsulfonamido)phenyl)-4-methoxyphenylsulfonamido) Acetate **47**

Prepared analogously to compound **25** from **45** (1.2 g, 1.88 mmol) and bromoacetonitrile (163 μL, 2.34 mmol). Flash chromatography (EtOAc/hexane 4:6 v/v) afforded **47** (800 mg, 62%) as a white solid. ^1^H NMR (400 MHz, DMSO-*d*_6_): δ (ppm) 7.65–7.59 (m, 2H), 7.50–7.41 (m, 3H), 7.36–7.30 (m, 3H), 7.15–7.06 (m, 4H), 6.89–6.83 (m, 4H), 4.90 (s, 2H), 4.67 (s, 2H), 4.32 (s, 2H), 4.06 (q, *J* = 7.1 Hz, 2H), 3.86 (s, 3H), 3.75 (s, 3H), 1.14 (t, *J* = 7.1 Hz, 3H). ^13^C NMR (100 MHz, DMSO-*d*_6_): δ (ppm) 168.8, 163.3, 162.5, 154.7, 139.5, 135.6, 133.5, 130.8, 129.9, 129.0, 128.2, 128.1, 127.7, 126.8, 126.7, 119.2, 116.2, 114.6, 114.0, 112.4, 69.6, 60.7, 55.8, 55.5, 50.5, 13.9.

##### Ethyl *N*-(3-(Benzyloxy)-4-((*N*-(cyanomethyl)-4-methoxyphenyl)sulfonamido)phenyl)-*N*-((4-methoxyphenyl)sulfonyl)glycinate **48**

Prepared analogously to compound **25** from **46** (1.8 g, 3.03 mmol) and ethyl bromoacetate (420 μL, 3.79 mmol). Flash chromatography (EtOAc/hexane 3:7 v/v) afforded **48** (1.2 g, 58%) as an off-white solid. ^1^H NMR (500 MHz, DMSO-*d*_6_): δ (ppm) 7.62 (d, *J* = 8.9 Hz, 2H), 7.51 (d, *J* = 8.9 Hz, 2H), 7.36–7.30 (m, 3H), 7.25–7.22 (m, 1H), 7.14 (dd, *J* = 6.5, 2.7 Hz, 2H), 7.09 (d, *J* = 8.9 Hz, 2H), 6.94–6.79 (m, 4H), 4.77–4.63 (m, 4H), 4.53 (s, 2H), 4.08 (q, *J* = 7.1 Hz, 2H), 3.84 (s, 3H), 3.77 (s, 3H), 1.14 (t, *J* = 7.1 Hz, 3H). ^13^C NMR (125 MHz, DMSO-*d*_6_): δ (ppm) 168.5, 162.9, 154.8, 141.6, 135.7, 131.8, 129.7, 129.6, 129.4, 128.2, 127.7, 127.0, 124.7, 119.4, 116.8, 114.4, 114.2, 112.3, 69.8, 60.9, 55.7, 55.6, 51.8, 38.4, 13.9.

##### Ethyl 2-(*N*-(4-(*N*-((1*H*-Tetrazol-5-yl)methyl)-4-methoxyphenylsulfonamido)-2-(benzyloxy)phenyl)-4-methoxyphenylsulfonamido)acetate **49**

To a solution of **47** (800 mg, 1.18 mmol) in DMF (15 mL) were added NaN_3_ (195 mg, 3.01 mmol) and NH_4_Cl (204 mg, 3.81 mmol), and the reaction was stirred at 100 °C for 24 h. On completion, the reaction mixture was diluted with H_2_O (50 mL), extracted with EtOAc (3 × 50 mL), and the combined organic layers were washed with H_2_O (3 × 150 mL) and sat. brine (3 × 150 mL), dried over anhydrous MgSO_4_, and evaporated to dryness under reduced pressure. The crude product was re-suspended in boiling MeOH and filtered to afford **49** (612 mg, 72%) as a white solid. ^1^H NMR (400 MHz, CDCl_3_): δ (ppm) 13.89–13.35 (br, 1H), 7.62–7.50 (m, 3H), 7.48–7.42 (m, 2H), 7.32–7.27 (m, 3H), 7.06–6.99 (m, 2H), 6.98–6.91 (m, 2H), 6.79 (d, *J* = 2.3 Hz, 1H), 6.73–6.67 (m, 2H), 6.54 (dd, *J* = 8.5, 2.3 Hz, 1H), 5.05 (s, 2H), 4.61 (s, 2H), 4.32 (s, 2H), 4.15 (q, *J* = 7.1 Hz, 2H), 3.87 (s, 3H), 3.76 (s, 3H), 1.23 (t, *J* = 7.1 Hz, 3H). ^13^C NMR (100 MHz, CDCl_3_): δ (ppm) 169.5, 164.0, 163.0, 155.3, 140.5, 135.4, 134.7, 131.4, 130.2, 129.5, 128.5, 128.2, 128.0, 127.4, 127.1, 119.2, 114.7, 114.0, 113.9, 70.4, 61.5, 55.9, 55.6, 50.9, 45.2, 14.2.

##### Ethyl *N*-(4-((*N*-((1*H*-Tetrazol-5-yl)methyl)-4-methoxyphenyl)sulfonamido)-3-(benzyloxy)phenyl)-*N*-((4-methoxyphenyl)sulfonyl)glycinate **50**

Prepared analogously to compound **49** from **48** (500 mg, 0.74 mmol) and NaN_3_ (120 mg, 1.85 mmol). Flash chromatography (DCM/EtOAc/formic acid 90:9:1 v/v/v) afforded **50** (219 mg, 41%) as a white solid. ^1^H NMR (500 MHz, DMSO-*d*_6_): δ (ppm) 7.55–7.49 (m, 2H), 7.47 (d, *J* = 8.9 Hz, 2H), 7.40–7.29 (m, 3H), 7.24 (d, *J* = 9.0 Hz, 1H), 7.14–7.03 (m, 4H), 6.89 (d, *J* = 8.9 Hz, 2H), 6.76 (dd, *J* = 6.2, 2.3 Hz, 2H), 5.06 (s, 2H), 4.60 (s, 2H), 4.49 (s, 2H), 4.08 (q, *J* = 7.1 Hz, 2H), 3.86 (s, 3H), 3.78 (s, 3H), 1.14 (t, *J* = 7.1 Hz, 3H). ^13^C NMR (125 MHz, DMSO-*d*_6_): δ (ppm) 168.5, 162.8, 162.7, 154.7, 141.1, 135.7, 133.1, 130.2, 129.6, 129.3, 129.2, 128.2, 127.7, 126.8, 124.8, 119.3, 114.3, 114.1, 112.1, 69.7, 60.9, 55.7, 55.5, 51.8, 42.7, 13.9.

##### *N*-(4-((*N*-((1*H*-Tetrazol-5-yl)methyl)-4-methoxyphenyl)sulfonamido)-2-(benzyloxy)phenyl)-*N*-((4-methoxyphenyl)sulfonyl)glycine **51**

Prepared analogously to compound **10** from **49** (560 mg, 0.78 mmol). Flash chromatography (EtOAc/hexane/formic acid 39:60:1 v/v/v) afforded **51** (400 mg, 74%) as a white solid. ^1^H NMR (500 MHz, DMSO-*d*_6_): δ (ppm) 13.22–12.25 (br, 2H), 7.58 (d, *J* = 8.9 Hz, 2H), 7.39–7.31 (m, 6H), 7.12 (d, *J* = 8.9 Hz, 2H), 7.06–7.01 (m, 2H), 6.87–6.75 (m, 4H), 5.14 (s, 2H), 4.53 (s, 2H), 4.20 (s, 2H), 3.86 (s, 3H), 3.75 (s, 3H). ^13^C NMR (125 MHz, DMSO-*d*_6_): δ (ppm) 170.3, 163.1, 162.4, 154.4, 139.9, 135.7, 133.3, 130.7, 129.4, 128.2, 128.1, 127.7, 126.1, 126.5, 120.3, 114.5, 113.9, 112.9, 69.6, 55.8, 55.5, 50.5, 44.3. HRMS (ESI): calcd for C_31_H_30_N_6_O_9_S_2_ [M – H]^−^, 693.1437; found, 693.1402. HPLC: *t*_R_ = 12.16 min, purity: >95%.

##### *N*-(4-((*N*-((1*H*-Tetrazol-5-yl)methyl)-4-methoxyphenyl)sulfonamido)-3-(benzyloxy)phenyl)-*N*-((4-methoxyphenyl)sulfonyl)glycine **52**

Prepared analogously to compound **10** from **50** (200 mg, 0.28 mmol). Flash chromatography (DCM/EtOAc/formic acid 90:9:1 v/v/v) afforded **52** (39 mg, 20%) as a white solid. ^1^H NMR (500 MHz, DMSO-*d*_6_): δ (ppm) 13.05–12.60 (br, 2H), 7.55–7.44 (m, 4H), 7.36–7.29 (m, 3H), 7.20 (d, *J* = 8.4 Hz, 1H), 7.07 (d, *J* = 8.8 Hz, 4H), 6.87 (d, *J* = 8.7 Hz, 2H), 6.80–6.70 (m, 2H), 5.03 (s, 2H), 4.59 (s, 2H), 4.37 (s, 2H), 3.82 (s, 3H), 3.77 (s, 3H). ^13^C NMR (125 MHz, DMSO-*d*_6_): δ (ppm) 169.9, 162.8, 162.6, 154.7, 141.2, 135.5, 133.1, 129.6, 129.4, 129.2, 128.2, 127.7, 126.9, 124.7, 119.2, 114.3, 114.1, 112.0, 69.7, 55.7, 55.5, 51.6, 42.8. HRMS (ESI): calcd for C_31_H_30_N_6_O_9_S_2_ [M + H]^+^, 693.1437; found, 693.1421. HPLC: *t*_R_ = 9.35 min, purity: >95%.

##### 3,5-Bis[(4-methoxyphenyl)sulfonamido]benzoic Acid **54**

To a solution of 3,5-diaminobenzoic acid **53** (1.0 g; 6.60 mmol) in DMF (5 mL) were added 4-methoxybenzenesulfonyl chloride (3.0 g, 14.52 mmol), pyridine (1.3 mL, 16.50 mmol), and DMAP (80 mg, 0.66 mmol), and the reaction was stirred at 100 °C overnight. On completion, the reaction was cooled to rt and diluted with EtOAc (100 mL). The organic layer was washed with 1 N HCl (3 × 50 mL), H_2_O (3 × 50 mL), and sat. brine (3 × 50 mL), dried over anhydrous MgSO_4_, and evaporated to dryness under reduced pressure to afford **54** (1.73 g, 53%) as a white solid. LC–MS *m*/*z* (ESI): 490.95 [M – H]^−^, *t*_R_ = 5.36 min, purity: >95%.

##### 3,5-Bis[(4-methoxyphenyl)sulfonamido]-*N*-phenylbenzamide **55**

To a solution of **54** (250 mg, 0.51 mmol) in DMF (5 mL) were added aniline (55 μL, 0.61 mmol), EDCI·HCl (245 mg, 1.28 mmol), and DMAP (125 mg, 1.02 mmol), and the reaction mixture was stirred at rt overnight. On completion, the solution was diluted with EtOAc (10 mL), washed with 1 N HCl (3 × 10 mL), sat. NaHCO_3_ (3 × 10 mL), and sat. brine (3 × 10 mL). The organic layer was dried over anhydrous MgSO_4_ and evaporated to dryness under reduced pressure to afford **55** (179 mg, 63%) as a white solid. LC–MS *m*/*z* (ESI): 566.05 [M – H]^−^, *t*_R_ = 6.19 min, purity: >95%.

##### *N*-Ethyl-3,5-bis[(4-methoxyphenyl)sulfonamido]benzamide **56**

Prepared analogously to **55** from **54** (250 mg, 0.51 mmol), ethylamine hydrochloride (83 mg, 1.02 mmol), EDCI·HCl (245 mg, 1.28 mmol), and DMAP (125 mg, 1.02 mmol) to afford **56** as a white solid (150 mg, 60%). LC–MS *m*/*z* (ESI): 519.00 [M – H]^−^, *t*_R_ = 5.43 min, purity: >95%.

##### *N*-Benzyl-3,5-bis[(4-methoxyphenyl)sulfonamido]benzamide **57**

Prepared analogously to **55** from **54** (250 mg, 0.51 mmol), benzylamine (110 μL, 1.02 mmol), EDCI·HCl (245 mg, 1.28 mmol), and DMAP (125 mg, 1.02 mmol) to afford **57** as a white solid (255 mg, 88%). LC–MS *m*/*z* (ESI): 580.05 [M – H]^−^, *t*_R_ = 6.10 min, purity: >95%.

##### 2,2′-((5-(Phenylcarbamoyl)-1,3-phenylene)bis(((4-methoxyphenyl)sulfonyl)azanediyl))diacetic Acid **58**

To a solution of **55** (180 mg, 0.32 mmol) in DMF (2 mL) was added K_2_CO_3_ (133 mg, 0.96 mmol) and ethyl bromoacetate (90 μL, 0.79 mmol) and the reaction mixture was stirred at rt overnight. On completion, the solution was diluted with H_2_O (30 mL), acidified to pH 5 with 2 M HCl, and extracted with EtOAc (4 × 20 mL). The combined organic layers were washed with H_2_O (3 × 50 mL) and sat. brine (3 × 50 mL), dried over anhydrous MgSO_4_, and evaporated to dryness under reduced pressure to afford the ester (220 mg, 96%) as a white solid that was used in the next step without further purification.

To a solution of the crude product (180 mg, 0.24 mmol) in THF/MeOH (10 mL, 1:1 v/v) was added a solution of NaOH (39 mg, 0.97 mmol) in H_2_O (5 mL), and the reaction mixture stirred at rt overnight. On completion, the reaction was acidified with 2 M HCl to pH 2, diluted with H_2_O (75 mL), and extracted with EtOAc (4 × 50 mL). The combined organic layers were washed with H_2_O (3 × 50 mL), sat. brine (3 × 50 mL), dried over anhydrous MgSO_4_ and evaporated to dryness under reduced pressure to afford **58** (148 mg, 83%) as a white solid. ^1^H NMR (400 MHz, DMSO): δ (ppm) 13.03–12.60 (br, 2H), 10.12 (s, 1H), 7.61 (d, *J* = 2.0 Hz, 2H), 7.57 (dd, *J* = 8.5, 1.0 Hz, 2H), 7.50–7.43 (m, 4H), 7.31–7.22 (m, 2H), 7.18 (t, *J* = 2.0 Hz, 1H), 7.06–6.99 (m, 1H), 6.98–6.90 (m, 4H), 4.27 (s, 4H), 3.73 (s, 6H). ^13^C NMR (101 MHz, DMSO): δ (ppm) 169.6, 163.5, 162.8, 140.5, 138.6, 135.6, 129.6, 129.5, 128.6, 125.4, 124.1, 120.8, 114.3, 55.5, 51.7, 15.2. HRMS (ESI): calcd for C_31_H_29_N_3_O_11_S_2_ [M – H]^−^, 682.1165; found, 682.1170. LC–MS *m*/*z* (ESI): 682.00 [M – H]^−^, *t*_R_ = 6.04 min, purity: >95%.

##### 2,2′-((5-(Ethylcarbamoyl)-1,3-phenylene)bis(((4-methoxyphenyl)sulfonyl)azanediyl))diacetic Acid **12**

Prepared analogously to **58** from **56** (155 mg, 0.28 mmol) to afford **12** as a white solid (113 mg, 61% over two steps). ^1^H NMR (400 MHz, DMSO): δ (ppm) 13.51–12.27 (br, 2H), 8.47 (t, *J* = 5.5 Hz, 1H), 7.59 (d, *J* = 2.0 Hz, 2H), 7.57–7.50 (m, 4H), 7.17 (t, *J* = 1.9 Hz, 1H), 7.07–7.02 (m, 4H), 4.31 (s, 4H), 3.83 (s, 6H), 3.24 (q, *J* = 7.2 Hz, 2H), 1.09 (t, *J* = 7.2 Hz, 3H). ^13^C NMR (101 MHz, DMSO): δ (ppm) 169.5, 164.0, 162.8, 140.3, 135.5, 129.5, 129.4, 128.0, 125.1, 114.3, 55.6, 51.6, 34.0, 14.6. HRMS (ESI): calcd for C_27_H_29_N_3_O_11_S_2_ [M – H]^−^, 634.1165; found, 634.1162. LC–MS *m*/*z* (ESI): 633.90 [M – H]^−^, *t*_R_ = 5.36 min, purity: >95%.

##### 2,2′-((5-(Benzylcarbamoyl)-1,3-phenylene)bis(((4-methoxyphenyl)sulfonyl)azanediyl))diacetic Acid **59**

Prepared analogously to **58** from **57** (225 mg, 0.39 mmol) to afford **59** as a white solid (185 mg, 68% over two steps). ^1^H NMR (400 MHz, DMSO): δ (ppm) 12.90 (s, 2H), 9.02 (t, *J* = 5.9 Hz, 1H), 7.63 (t, *J* = 3.5 Hz, 2H), 7.57–7.51 (m, 3H), 7.36–7.30 (m, 2H), 7.25 (ddd, *J* = 6.2, 5.3, 2.1 Hz, 3H), 7.07–7.02 (m, 3H), 4.44 (d, *J* = 5.8 Hz, 2H), 4.33 (s, 3H), 3.83 (s, 6H), 3.18 (s, 1H); ^13^C NMR (101 MHz, DMSO): δ (ppm) 169.6, 162.8, 162.8, 140.5, 139.2, 129.5, 129.4, 128.3, 127.1, 126.8, 125.2, 114.4, 55.7, 51.5, 42.3. HRMS (ESI): calcd for C_32_H_31_N_3_O_11_S_2_ [M – H]^−^, 696.1322; found, 696.1303. LC–MS *m*/*z* (ESI): 696.00 [M – H]^−^, *t*_R_ = 5.97 min, purity: >95%.

##### 2,2′-((5-(Benzylcarbamoyl)-1,3-phenylene)bis(((4-methoxyphenyl)sulfonyl)azanediyl))diacetamide **60**

Compound **57** (0.25 g, 0.43 mmol) was dissolved in DMF (2 mL) in a round-bottomed flask. K_2_CO_3_ (0.178 g, 3 equiv) was added with stirring for 3 min, followed by 2-bromoacetamide (0.148 g, 2.5 equiv). The reaction mixture was stirred overnight at rt and monitored by TLC. The solution was diluted in water (30 mL) and adjusted to pH 5 with 2 M HCl, then extracted with EtOAc (4 × 20 mL). The combined organic layers were washed with water (3 × 50 mL) then with brine (1 × 50 mL). The organic layer was dried over MgSO_4_, and the solvent was removed under vacuum. The residue was suspended in Et_2_O (3 mL) and then the solvent evaporated under vacuum. The residue was stirred with hexane (5 mL) overnight. The hexane was decanted carefully and the resulting solid **60** (0.278 g, 93%) was dried under vacuum. ^1^H NMR (400 MHz, DMSO): δ (ppm) 8.96 (t, *J* = 5.9 Hz, 1H), 7.68 (d, *J* = 2.0 Hz, 2H), 7.54–7.49 (m, 4H), 7.37–7.29 (m, 5H), 7.28–7.23 (m, 3H), 7.16 (s, 2H), 7.10–7.04 (m, 4H), 4.45 (d, *J* = 5.8 Hz, 2H), 4.11 (s, 4H), 3.83 (s, 6H); ^13^C NMR (101 MHz, DMSO): δ (ppm) 168.6, 164.4, 162.9, 140.5, 139.2, 135.0, 129.7, 128.9, 128.3, 127.2, 126.8, 125.6, 114.4, 55.7, 42.6. HRMS (ESI): calcd for C_32_H_33_N_5_O_9_S_2_ [M – H]^−^, 694.1641; found, 694.1633. LC–MS *m*/*z* (ESI): 694.00 [M – H]^−^, *t*_R_ = 5.57 min, purity: >95%.

##### *N*-Benzyl-3,5-bis{[*N*-(cyanomethyl)-4-methoxyphenyl]sulfonamido}benzamide **61**

Prepared analogously to **60** from **57** (0.25 g, 0.43 mmol) and bromoacetonitrile (0.128 g, 2.5 equiv) to afford **61** as an off-white solid (0.221 g, 88%). ^1^H NMR (400 MHz, DMSO): δ (ppm) 9.14 (t, *J* = 5.9 Hz, 1H), 7.79 (d, *J* = 2.0 Hz, 2H), 7.60–7.56 (m, 4H), 7.35 (ddd, *J* = 7.1, 4.4, 1.6 Hz, 2H), 7.31–7.23 (m, 3H), 7.21 (t, *J* = 2.0 Hz, 1H), 7.13–7.07 (m, 4H), 4.87 (s, 4H), 4.47 (d, *J* = 5.8 Hz, 2H), 3.84 (s, 6H); ^13^C NMR (101 MHz, DMSO): δ (ppm) 163.8, 163.4, 139.5, 139.1, 129.8, 128.3, 127.8, 127.3, 126.6, 114.8, 55.8, 42.7. HRMS (ESI): calcd for C_32_H_29_N_5_O_7_S_2_ [M + Cl]^−^, 694.1197; found, 694.1174. LC–MS *m*/*z* (ESI): 658.15 [M – H]^−^, *t*_R_ = 6.78 min, purity: >95%.

### Molecular Docking

The Keap1 Kelch domain protein coordinates were obtained from the PDB (ref: 4IQK); the ligand, water molecules, and ions were removed from the structure file and the protein structure was parameterized and saved in pdbqt format using AutoDockTools. The small molecules were prepared in 2D format using ChemDraw and then converted into 3D coordinates using Chem3D (ChemOffice 19, PerkinElmer). The ligands were energy minimized using the MM2, MMFF94, and Mopac (PM7) protocols with default parameters and then saved as mol2 files. The mol2 files were converted into pdbqt files using openbabel 2.3.2.^41^ The compounds were docked to the Keap1 Kelch domain using qvina 2.1^42^ with default parameters and an exhaustiveness setting of 10. The best docked conformations were saved and analyzed in UCSF Chimera 1.14.^43^ Images of the docked structures and protein conformers were prepared in UCSF Chimera, LigPlot Plus,^44^ and Pymol (The PyMOL Molecular Graphics System, Version 2.0 Schrödinger, LLC).

### Sub-Cloning, Expression, and Purification of the Keap1 Kelch Domain

The coding sequence for the Keap1 Kelch domain was sub-cloned into a modified pEt15b vector with a His-SUMO tag and Ulp1 cleavage site. The plasmid was transformed into *Escherichia coli* BL21 CodonPlus cells (Novagen) and grown in 6 L of the Terrific Broth (TB) medium at 37 °C, supplemented with 100 mg/L ampicillin, to an *A*_600_ of 1.0 and induced for 24 h at 20 °C with 0.5 mM isopropyl β-d-thiogalactoside (IPTG; Melford). Cells were harvested by spinning them down in a Beckman centrifuge using a JLA 16.25 rotor at 6000 rpm for 20 min. Protein purification was carried out in three steps. The cells were initially resuspended in lysis buffer (50 mM HEPES, pH 7.4, 300 mM NaCl and 10 mM imidazole) and sonicated at 16 μm amplitude for 10 cycles of 30 s ON and 45 s OFF. The lysate was then cleared by centrifugation at 20,000 rpm at 4 °C using a JA 25.5 rotor. To perform the first affinity chromatography, the lysate was loaded onto a 5 mL His-Trap column (GE Healthcare, UK) pre-equilibrated with lysis buffer, washed for 50 column volumes of wash buffer (lysis buffer supplemented with 50 mM imidazole), and eluted with elution buffer (lysis buffer supplemented with 250 mM imidazole). The purified protein was pooled and cleaved overnight with UlP1 protease supplemented with 3 mM DTT. Simultaneous with the cleavage, the protein was also dialyzed against buffer containing 50 mM HEPES, pH 7.4, and 300 mM NaCl. Thereafter, the protein was passed through the His-Trap column for the second time using the same buffers as before. The cleaved protein without the His-SUMO tag was captured in the flow, which was then pooled and passed through a 16/60 Superdex 200 pg size exclusion column with a buffer containing 50 mM HEPES, pH 7.5, and 200 mM NaCl to further purify Keap1. Lastly, the protein was then pooled and concentrated to 10 mg/mL using an Amicon ultraconcentration device (Millipore), aliquoted, flash-frozen in liquid nitrogen, and stored at −80 °C for subsequent use.

### FP Assays

The FP assays were carried out according to previously described methods.^8,9,12,45^ Briefly, varying concentrations of the small molecule inhibitor dissolved in DMSO were plated into untreated Corning black 96 well plates containing a solution of the Keap1 Kelch domain (200 nM final concentration) and the fluorescent peptide FITCβ-DEETGEF-OH (1 nM final concentration) in Dulbecco’s phosphate-buffered saline (DPBS) at pH 7.4 (11% final DMSO concentration, 100 μL final volume). Following incubation under slow agitation (30 min at RT in the absence of light), the plates were transferred to a PHERAstar microplate reader and the FP was recorded. All measurements were recorded in triplicate. The data were normalized to the control and then fitted to a standard four-parameter logistic function using Origin Pro software.

### DSF Assays

Solutions of the Keap1 Kelch domain protein (5 μM final concentration) and the small molecule inhibitor (10 μM final concentration) were prepared in DPBS at pH 7.4 (10% final DMSO concentration, 30 μL final volume) in an Eppendorf tube. The solution was centrifuged briefly (1500 rpm, 60 s) to remove any particulates and mix the samples, followed by a 30 min incubation at RT. The samples were loaded into Tycho NT.6 capillaries (Nanotemper) in triplicate and positioned in the Tycho NT 1.6 instrument. The samples were heated from 35 °C to 95 °C using the default settings and the ratio of the intrinsic tyrosine and tryptophan fluorescence intensities (350 and 330 nm) for each capillary tube was recorded. The fluorescence ratio versus temperature was plotted and the inflection temperature (*T*_i_) of the profile was calculated and recorded within the instrument software. Changes in inflection (Δ*T*_i_) for the inhibitors were calculated by subtracting the mean *T*_i_ of the Keap1 sample plus vehicle (*n* = 6) from the mean Keap1 plus inhibitor value (*n* = 3).

### ITC Experiments

The measurements were performed as described previously.^45^ Briefly, the protein was dialyzed overnight against buffer containing 25 mM HEPES, pH 7.4, and 200 mM NaCl and concentrated to 50 μM in the presence of 5% DMSO to match the ligand solution. The small molecule inhibitor was diluted to a concentration of 500 μM in the dialysis buffer (5% max final concentration of DMSO). ITC experiments were performed with a MicroCal PEAQ-ITC instrument (Malvern Instruments, UK). Titrations were carried out at 25 °C with a stirring speed of 750 rpm. The inhibitor was titrated into the cell containing the protein solution over 30 injections with the first injection of 0.3 μL, followed by 29 injections of 1.3 μL and a gap of 120 s between each injection. Data analyses were performed as previously described. For each experiment, at least two titrations were performed. Titration data were analyzed independently, and the obtained values were averaged.

### Crystallization and Structure Determination of the Keap1–Compound **11** Complex

Crystals for the Keap1 Kelch domain in complex with **11** appeared after 2–3 days in 3.7 M sodium formate, pH 7.0 at 18 °C. The crystals were further optimized by streak seeding to obtain single crystals for diffraction measurements. The single crystals of the complex were cryo-protected with 20% w/v ethylene glycol in 1.2-fold of the crystallization solution and flash-frozen in liquid nitrogen.

Diffraction data for individual crystals were collected at beamlines I03 at Diamond Light Source. Data were processed using either XDS^46^ or iMosflm^47^ and scaled to resolutions as mentioned in Table S4.^48^ The structure of the complex was solved by molecular replacement using the native Keap1 structure (PDB entry 1ZGK)^49^ as a search model. The crystallographic statistics are given in Table S4. Crystals of the binary Keap1 Kelch domain in complex with **11** contain one molecule in the asymmetric unit.

### Solubility and PAMPA Assays

Solubility measurements were performed using Multiscreen HTS-PCF filter plates (Merck Millipore, MSSLBPC10) at pH 7.4 according to the manufacturer’s instructions. Membrane permeability was evaluated using the PAMPA using the hexadecane-method PAMPA protocol with HDM-PAMPA multiscreen permeability filter plates (Merck Millipore, MAIPNTR10) according to the manufacturer’s instructions.

### Biomimetic HPLC Methods

The chromatographic measurements of CHIlogD (CHI) were carried out using the compounds’ calibrated gradient retention times obtained from an Agilent 1100 HPLC fitted with a Gemini NX-C-18 column (Phenomenex Ltd Macclesfield, UK) with dimensions of 50 × 3 mm and 5 μm particle size. The mobile phase A was either 0.01 M formic acid (pH 2.6), a 50 mM ammonium acetate buffer with an adjusted pH of 7.4, or a 50 mM ammonium acetate buffer with an adjusted pH of 10.5. The mobile phase B was 100% acetonitrile. The flow rate was 1.0 mL/min. An acetonitrile linear gradient was used from 0 to 100%. The acetonitrile concentration reached 100% in 3.5 min. The 100% acetonitrile mobile phase was maintained for an additional 1 min before it was returned to 0% at 4.7 min. The gradient run cycle time was 6 min, with an additional equilibration time of 1 min before the next injection. The gradient retention times were calibrated using the CHI values of reference compounds.

The phospholipid-binding was measured using an IAM PC.DD2 column with dimensions of 100 × 4.6 mm (Regis Technologies Inc., Morton Grove, IL, USA). The gradient retention times were measured using a 50 mM ammonium acetate mobile phase with the pH adjusted to 7.4. The mobile phase flow rate was 1.5 mL/min. The acetonitrile gradient was applied to reach 90% in 4.75 min. The 90% acetonitrile concentration was maintained for an additional 0.5 min (to 5.25 min) and then returned to 0% by 5.5 min. The cycle time was 6 min, plus an additional 1 min equilibration time was applied while the injector prepared for the next injection. The gradient retention times were calibrated with the acetophenone homologues for which the CHI IAM values have been established using isocratic measurements.

The protein binding measurements were carried out on Chiralpak HSA and Chiralpak AGP columns with dimensions of 3 × 50 mm and 5 μm particle size (Chiral Technologies Europe, France). The mobile phase was 50 mM ammonium acetate adjusted to pH 7.4, with a 1.2 mL/min flow rate. The standard isopropanol gradient reached 35% in 3.5 min, which was maintained for 1 min, before returning to 0% at 4.7 min. The cycle time was 6 min with an additional 1 min re-equilibration time. The racemic warfarin showed separation of its enantiomers. The retention times were calibrated using literature protein binding data of nine marketed drugs.

### Cytotoxicity Assay

Retinal pigment epithelial ARPE19 cells (non-carcinoma cells) were grown and maintained in a DMEM F12 medium supplemented with 10% fetal bovine serum and 2 mM l-glutamine at 37 °C, 5% CO_2_. The cells were plated in 96-well culture plates at a density of 10,000 cells per mL and allowed to adhere at 37 °C for 24 h. The following day, various doses of drugs or vehicle were added to the cells and further incubated for 96 h. Then, the supernatant was removed and MTT [3-(4,5-dimethylthiazol-2-yl)-2,5-diphenyltetrazolium bromide] was added for 4 h. The ability of cells to form formazan crystals by active mitochondrial respiration was determined by using a Microplate reader after dissolving the crystals in DMSO. Cytotoxicity was expressed as a relative percentage of the absorbance measured at 540 nm in the control and drug-treated cells.

### Real-Time PCR

Hepa1c1c7 cells were seeded at 5 × 10^5^ cells/well of six-well plates. On the following day, cells were treated with compound **11** (50 μM) or vehicle (0.1% DMSO) for 16 h, in triplicates. Cells were then washed twice in PBS, lysed, RNA was extracted, and cDNA was synthesized using standard methods. The levels of mRNA for each gene were determined by real-time quantitative PCR (TaqMan) and normalized for the levels of 18S mRNA.

### Cellular Thermal Shift Assay

HL-60 cells (3 × 10^7^) grown in RPMI 1640 (Invitrogen) supplemented with 10% (v/v) heat-inactivated fetal bovine serum (Invitrogen) were pelleted by centrifugation at 300*g* for 4 min at room temperature (RT), washed with phosphate-buffered saline (PBS) twice, resuspended in PBS (3 mL) containing 1× protease inhibitor cocktail (Roche), and snap frozen in liquid N_2_. Cell lysates were obtained by four freeze–thaw cycles and cell debris were removed by centrifugation at 17,000*g* for 15 min at 4 °C. The supernatant (lysate) was split into two tubes, each containing 1.3 mL of lysate, and incubated with either 0.1% (v/v) DMSO or 50 μM of compound **11** at 37 °C for 1 h. Following incubation, 100 μL of lysate was aliquoted into 12 PCR tubes and subjected to various temperatures (38–60 °C) for 3 min using the VeriFlex blocks in the Veriti 96-well thermal cycler (Thermo Fisher Scientific). After cooling for 3 min at 25 °C, the samples were snap frozen. On the following day, the samples were thawed out at 25 °C, and the insoluble fractions were removed by centrifugation at 17,000*g* for 40 min at 4 °C. The soluble fractions (60 μL) were transferred to fresh microcentrifuge tubes containing 20 μL of 4× LDS buffer (Thermo Fisher Scientific) and 8 μL of Sample Reducing Agent (Thermo Fisher Scientific), mixed, and incubated at RT for 30 min. Proteins were resolved by electrophoresis on 4–12% NuPAGE Bis-Tris gels (Thermo Fisher Scientific) with MOPS running buffer and transferred onto a 0.45 μm nitrocellulose membrane. The membrane was blocked in 5% (w/v) non-fat milk in PBS-0.1% Tween 20 (Milk-PBST) for 1 h, incubated overnight (16 h) at 4 °C with the primary rat monoclonal Keap1 antibody (1:2000 in Milk-PBST, MABS514, Millipore), washed thrice in PBS-0.1% (v/v) Tween 20 (PBST) for 30 min, and incubated with goat anti-rat 680/800 IRDye secondary antibody (1:20,000 in Milk-PBST) for 1 h at RT protected from light. The blots were washed thrice in PBST for 30 min before scanning using Odyssey (LI-COR) imager, where the Keap1 band fluorescence intensity for each condition was quantified and normalized to the 38 °C sample intensity.

## Supporting Information Available

X-ray crystallography statistics, biophysical assays, cell-based assays, and selected compound analytical data (PDF).
Molecular formula strings (CSV)

## Accession Codes

PDB ID of New Crystal (X-ray) Structures: Atomic coordinates and experimental data for compound **11** bound to the Keap1 Kelch domain are available: PDB entry 6HWS.

## Abbreviations

AGP: α-glycoprotein
ARE: antioxidant response element
CDDO-Me: bardoxolone methyl
CETSA: cellular thermal shift assay
CHI: chromatographic hydrophobicity index
DE_max_: maximum drug efficiency percentage
DMF: dimethyl fumarate
DPBS: Dulbecco’s phosphate-buffered saline
DSF: differential scanning fluorimetry
FP: fluorescence polarization
GstP: glutathione *S*-transferase P
Gclc: glutamate–cysteine ligase catalytic subunit
Gclm: glutamate–cysteine ligase modulatory subunit
HEPES: 4-(2-hydroxyethyl)-1-piperazineethanesulfonic acid
Hmox1: heme oxygenase 1
IAM: immobilized artificial membrane
IPTG: isopropyl β-d-thiogalactoside
ITC: isothermal titration calorimetry
Keap1: Kelch-like ECH-associated protein 1
MOPS: 3-(*N*-morpholino)propanesulfonic acid
MTT: 3-(4,5-dimethylthiazol-2-yl)-2,5-diphenyltetrazolium bromide
Neh2: Nrf2–ECH homology 2 domain
NQO1: NAD(P)H quinone oxidoreductase 1 or NAD(P)H quinone dehydrogenase 1
Nrf2: nuclear factor erythroid 2-related factor 2
PPI: protein–protein interaction
Rbx1: ring-box 1
TB: Terrific Broth
*T*_i_: inflection temperature
Δ*T*_i_: change in inflection temperature
*V*_d_: volume of distribution
