# Supplementary material for: Phenyl Bis-Sulfonamide Keap1-Nrf2 Protein–Protein Interaction Inhibitors with an Alternative Binding Mode
Source: J Med Chem. 2022 May 12;65(10):7380–98. doi: 10.1021/acs.jmedchem.2c00457 (PMC9150106; doi:10.1021/acs.jmedchem.2c00457)

Phenyl *bis*-sulfonamide Keap1-Nrf2 protein-protein interaction inhibitors with an alternative binding mode

Nikolaos Georgakopoulos[1,2], Sandeep Talapatra[1], Dina Dikovskaya[3], Sharadha Dayalan Naidu[3], Maureen Higgins[3], Jemma Gatliff[1,2], Aysel Ayhan[1], Roxani Nikoloudaki[1,2], Marjolein Schaap[1], Klara Valko[1,4], Farideh Javid[5], Albena T. Dinkova-Kostova, Frank Kozielski[1] and Geoffrey Wells[1*]

[1]UCL School of Pharmacy, University College London, 29/39 Brunswick Square, London, WC1N 1AX, UK, [2]Keregen Therapeutics Ltd, Stevenage Bioscience Catalyst, Gunnels Wood Rd, Stevenage, SG1 2FX, UK, [3]Jacqui Wood Cancer Centre, Division of Cellular Medicine, University of Dundee School of Medicine, Dundee, DD1 9SY, Scotland, UK [4]Bio-Mimetic Chromatography Consultancy, 17 Cabot Close, Stevenage, SG2 0ES, UK. [5] Department of Pharmacy, University of Huddersfield, Queensgate, Huddersfield, HD1 3DH, UK, [6]Department of Pharmacology and Molecular Sciences and Department of Medicine, Johns Hopkins University School of Medicine, Baltimore, MD 21205, USA.

Corresponding Author
*E-mail: g.wells@ucl.ac.uk

Supplementary Data

Table of contents

Table S1 SAR and physicochemical properties for compounds **12** and **58** – **61**. Page S2
Table S2 Cytotoxicity of selected compounds against ARPE19 cells. Page S2
Table S3 Solubility and PAMPA permeability of selected compounds. Page S3
Table S4 Crystallographic statistics for the Keap1-Kelch-domain-**11** complex. Page S4
Figure S1 Representative ITC titration profiles for selected ligands. Page S5
Figure S2 Compound 11 increases the thermal stability of Keap1. Page S5
HPLC profiles of selected compounds. Pages S6-9

Table S1. Structure-activity relationships and physicochemical properties for compounds **12** and **58 – 61**.

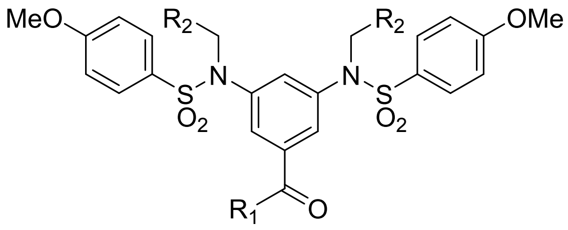

Compd	R_1	R_2	FP inhibition at 10 μM (%)	NQO1-fold induction at 10 μM[a]
**58**	NHPh	CO_2H	54%	0.82 ± 0.34
**12**	NHEt	CO_2H	< 25%	1.17 ± 0.14
**59**	NHBn	CO_2H	42%	1.21 ± 0.11
**60**	NHBn	$CONH_2$	< 25%	1.26 ± 0.07
**61**	NHBn	CN	< 25%	0.98 ± 0.14

Notes: a. Fold-induction of NQO1 enzymatic activity after 24 h treatment relative to the DMSO control.

Table S2. Cytotoxicity of selected compounds against ARPE19 cells.

**Compound**	**IC_{50} concentration (μM)**
**Sulforaphane**	9.89 ± 2.91
**4**	70% ± 7.05[a]
**11**	NI[b]

Notes: a. Percentage inhibition of cell growth at 100 μM; b. NI – no observed inhibition of cell growth at 100 μM.

Table S3. Solubility and PAMPA permeability of selected compounds.

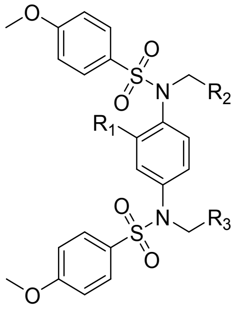

Compd	R_1	R_2	R_3	Solubility (μg/mL)	log*Pe*[a]
**10**	OMe	CO_2H	CO_2H	271	-4.74
**31**	OEt	CO_2H	CO_2H	337	-5.13
**32**	O*n*Bu	CO_2H	CO_2H	362	-4.94
**11**	OBn	CO_2H	CO_2H	348	-5.17
**33**	OPh	CO_2H	CO_2H	363	-4.78
**34**	Ph	CO_2H	CO_2H	298	-4.76
**36**	O*n*Pr	CO_2H	CO_2H	347	-4.70
**37**	O*i*Pr	CO_2H	CO_2H	288	-4.74
**38**	OBn(3-OMe)	CO_2H	CO_2H	379	-4.86
**39**	OH	CO_2H	CO_2H	343	-4.81
**40**	OBn	$CONH_2$	$CONH_2$	< 50	ND[b]
**41**	OBn	CN	CN	< 50	-4.19
**42**	OBn	TET	TET	< 50	ND[b]
**51**	OBn	CO_2H	TET	254	-5.19
**52**	OBn	TET	CO_2H	256	4.66

Notes: a. Permeability coefficient at pH 7.4; b. ND - not determined.

Table S4. Crystallographic statistics for the Keap1-Kelch-domain-**11** complex.

	**Keap1-Kelch-Compound 11**
**Data Collection**	
**Wavelength (Å)**	0.976
**Resolution range**	1.75 – 65 (1.75 – 1.84)
**Space group**	C2
**Unit cell (a, b, c, α, β, γ)**	125.96, 75.74, 48.88, 90, 106.43, 90.00
**Total reflections**	179698 (25338)
**Unique reflections**	43651 (6314)
**Multiplicity**	4.1 (4.0)
**Completeness (%)**	98.4 (97.6)
**Mean I/σ (I)**	9.0 (2.1)
**R_{merge}**	9.7 (60.8)
**Wilson B-factor (Å^2)**	18.6
**Average B-factor (Å^2)**	
**Overall**	23.3
**Protein**	20.5
**Ligand**	33.1
**Solvent**	36.0
**R_{work}/ R_{free}**	15.8 / 19.3
**R.m.s.d., bonds**	0.013
**R.m.s.d., angles**	1.21
**Ramachandran plot**	
**..Favored (%)**	97.2
**..Allowed (%)**	2.82
**..Outliers (%)**	0.01

Statistics for the highest-resolution shell are shown in parentheses.

Figure S1 Representative isothermal titration calorimetry (ITC) profiles for selected ligands.

Compound 11 | Compound 31 | Compound 32 | Compound 36

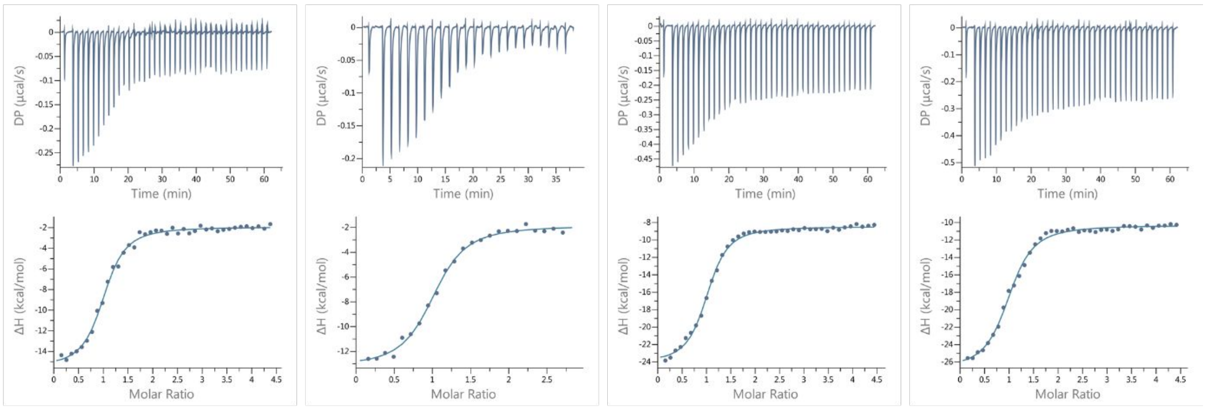

Compound 38

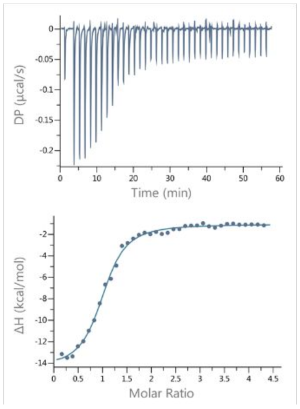

Compound	K_d (nM)	ΔG (kcal/mol)	ΔH (kcal/mol)	-TΔS (kcal/mol)
**11**	500 ± 57.4	-8.60	-13.7 ± 0.275	5.07
**31**	429 ± 61	-8.69	-11.4 ± 0.299	2.76
**32**	450 ± 39.3	-8.66	-15.7 ± 0.232	7.07
**36**	638 ± 68.4	-8.45	-16.6 ± 0.349	8.15
**38**	541 ± 54.7	-8.55	-13.4 ± 0.255	4.88

Figure S2. Compound **11** increases the thermal stability of Keap1.

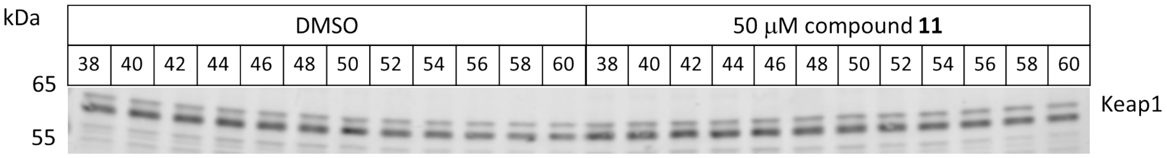

HL-60 cell lysates were incubated with vehicle (0.1% DMSO) or compound **11** (50 µM) for 1 h at 37°C . Subsequently, the treated cell lysates were heated at various temperatures (38-60°C, at increments of 2°C) for 3 minutes, and the insoluble fractions were removed by centrifugation. The protein levels of Keap1 in each of the soluble fractions were determined by western blot analysis.

HPLC profiles of selected compounds

Compound 11

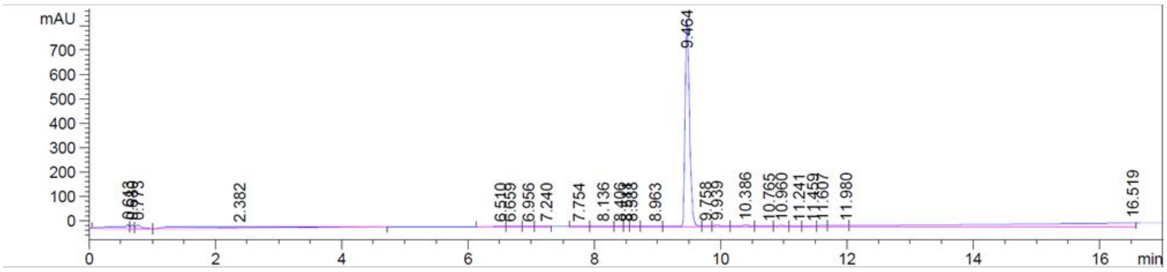

Compound 31

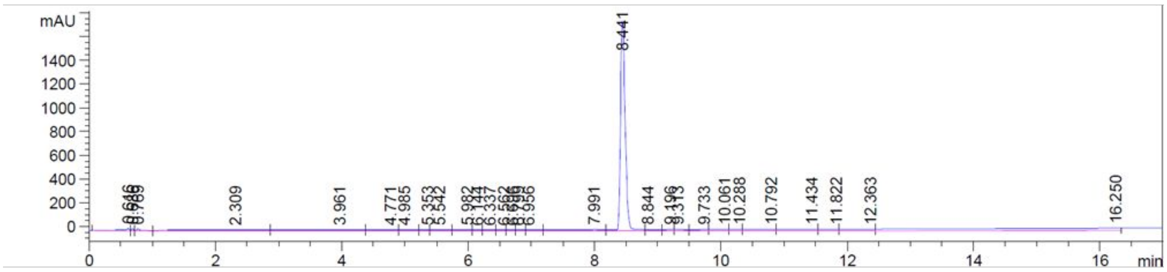

Compound 32

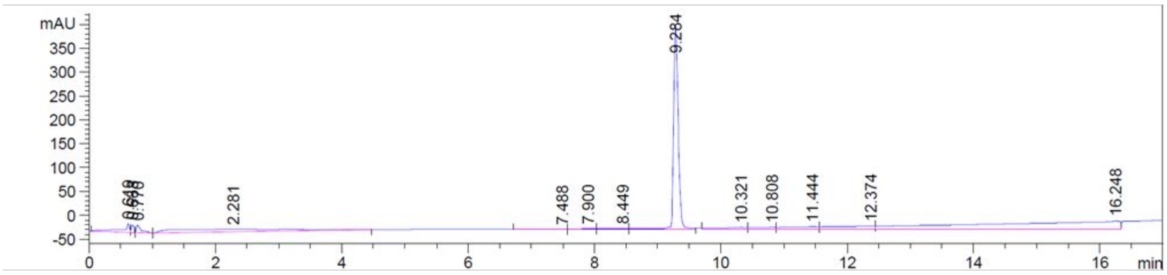

Compound 33

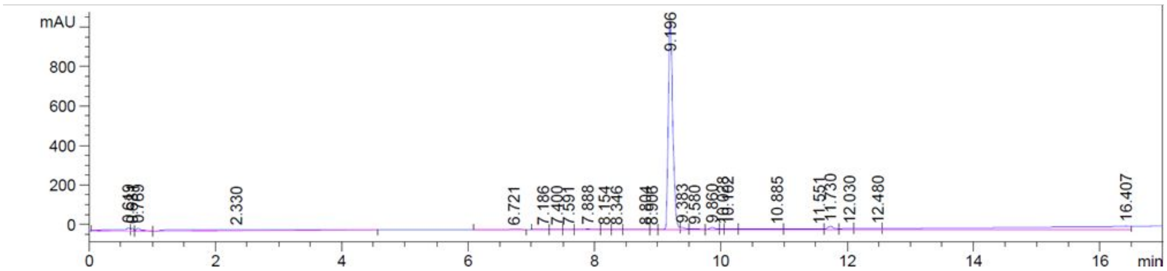

Compound 34

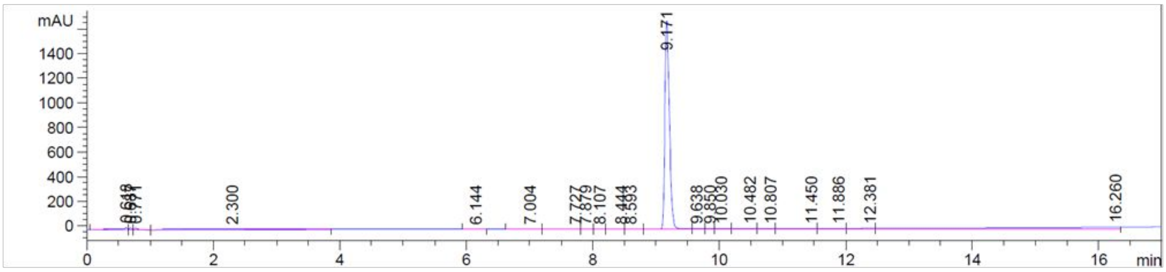

Compound 36

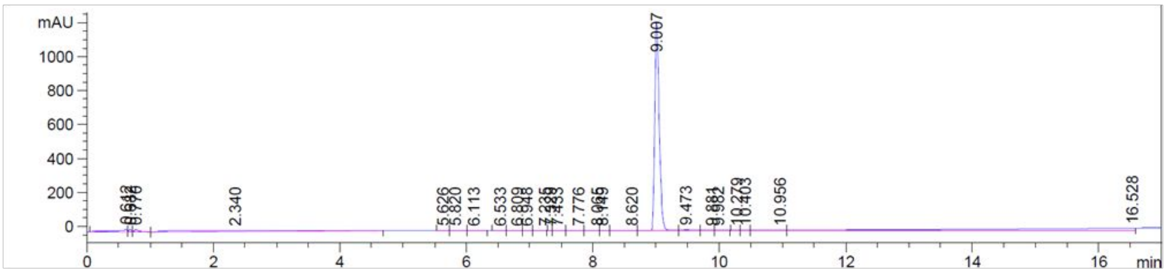

Compound 37

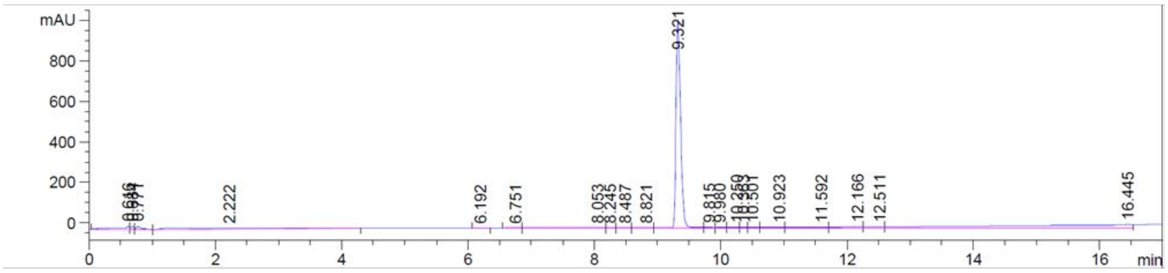

Compound 38

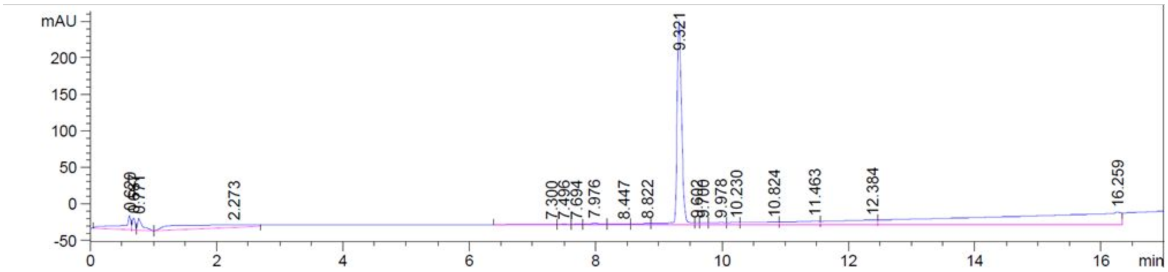

Compound 39

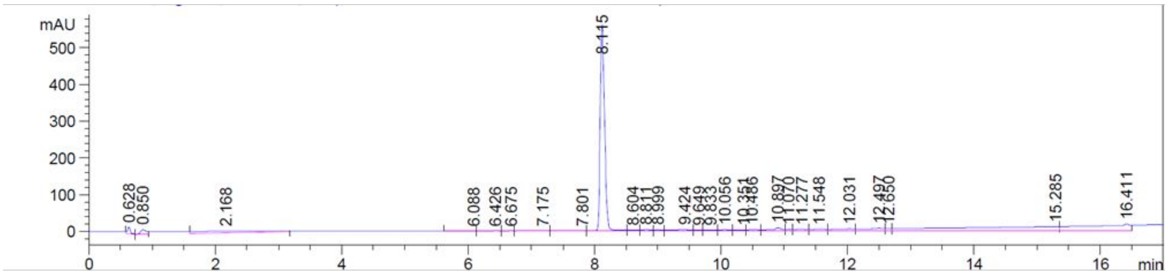

Compound 40

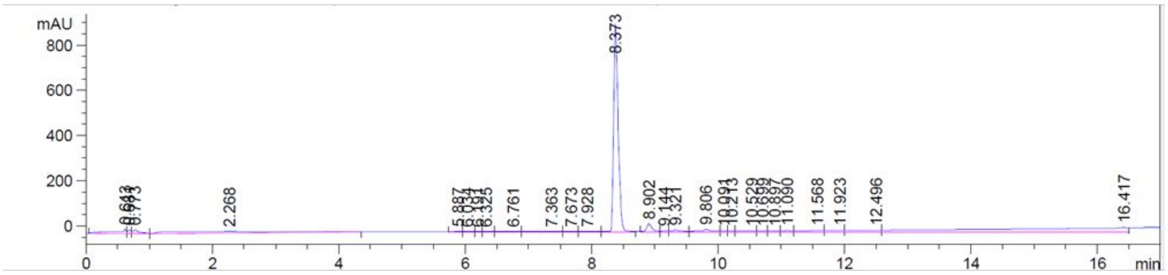

Compound 41

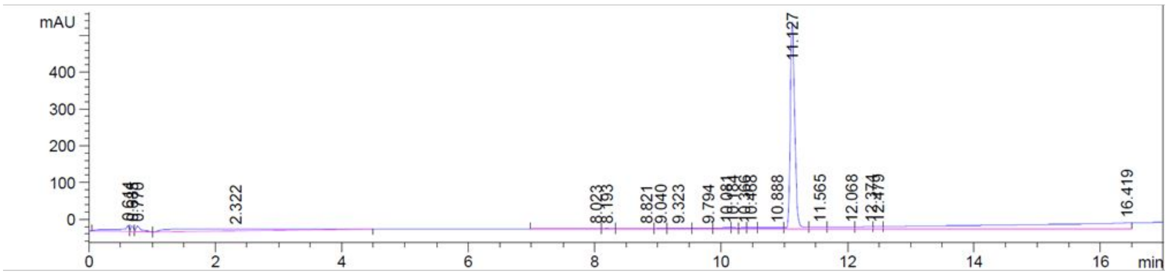

Compound 42

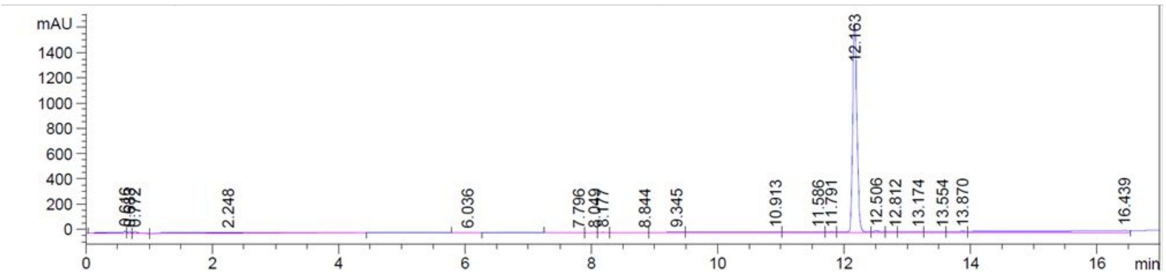

Compound 51

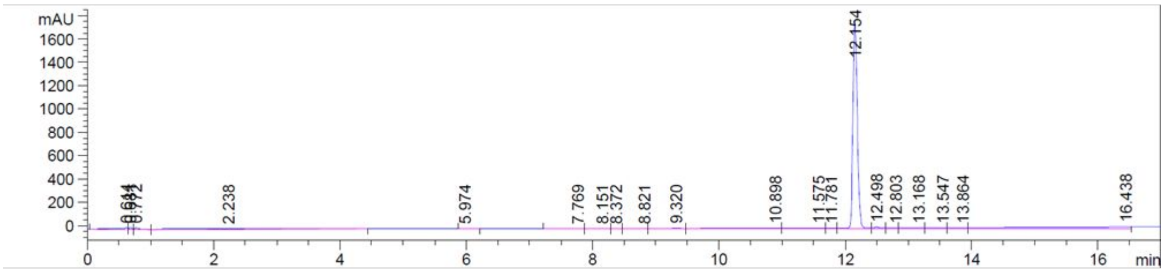

Compound 52

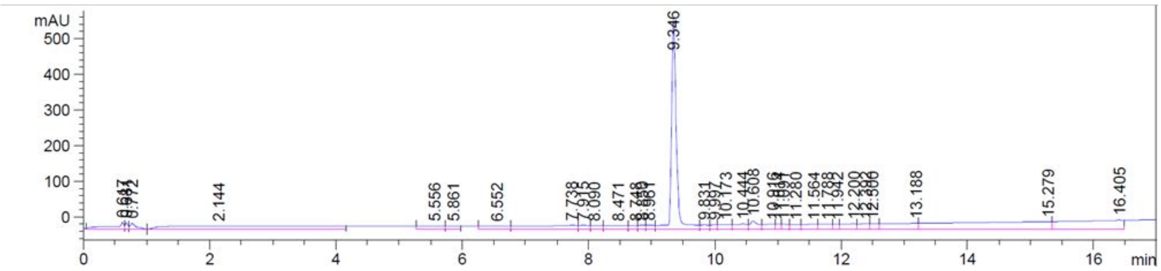

Supplement: Supplementary file 1 — jm2c00457_si_001.pdf [file jm2c00457_si_001.pdf]
